# Assessing the Feasibility of Bioscrubbing for Flue Gas Treatment and Sulfur Recovery: A Comparative Study Using Mathematical Modeling, Life Cycle Analysis, and Life Cycle Costing

**DOI:** 10.1021/acsenvironau.5c00216

**Published:** 2025-12-22

**Authors:** Alessio Castagnoli, Eric Valdés, Francesco Pasciucco, Isabella Pecorini, Daniel González Alé, Giulio Munz, David Gabriel

**Affiliations:** † Department of Energy, Systems Territory and Construction Engineering, 9310 University of Pisa, Via C.F. Gabba 22, Tuscany, 56122 Pisa, Italy; ‡ GENOCOV Research group, Department of Chemical, Biological and Environmental Engineering, 323882 Escola d’Enginyeria, Universitat Autònoma de Barcelona, 08193 Bellaterra, Spain; § Department of Civil and Environmental Engineering, 9300 University of Florence, Via di S. Marta, 3, 50139 Firenze, Italy

**Keywords:** bioscrubber, desulfurization, mathematical modeling, ReCiPe 2016 (H), net present value (NPV), circular bioeconomy

## Abstract

Industrial flue gas emissions are treated with technologies such as wet flue gas desulfurization (FGD) in chemical scrubbers, which are costly. Two-step biological scrubbers have emerged as an alternative for bio-FGD. However, no holistic technoeconomic and environmental comparison of both approaches is yet available. This study evaluates a conventional chemical scrubber (CS) and a bioscrubber (BS) treating sulfur-rich off-gas from a sulfur-based pigment plant. The bioscrubber integrates anaerobic sulfate reduction and partial sulfide oxidation to recover elemental sulfur and biogas. Two BS variants were analyzed, differing in carbon source for sulfate reduction: fossil-derived pure glycerin (BS-PG) and purified crude glycerol (BS-PCG). Mathematical models were integrated with life cycle assessment (LCA) and life cycle costing (LCC). Bioscrubbing enables resource recovery but strongly depends on the carbon source: BS-PG raises environmental impacts in most categories and increases greenhouse gas emissions to about 7277 tCO_2_eq per year, compared with 1379 tCO_2_eq for CS, whereas BS-PCG limits them to 1599 tCO_2_eq and performs better than CS in several impact categories. Nonetheless, the energy and chemical demands for glycerol purification remain challenging. Sensitivity analyses identified gas flow rate, purge fraction, and distance to disposal sites as crucial parameters, indicating that bioscrubbing may be suited for medium-to-small plants. Economic analysis indicates that carbon source purchase dominates costs (≈1.6 M€/year for BS-PG and 1.2 M€/year for BS-PCG), so feasibility hinges on lowering glycerol prices and valorizing biogas. Overall, the integrated assessment highlights key trade-offs and design levers for enhancing the sustainability and viability of bioscrubber systems.

## Graphical abstract

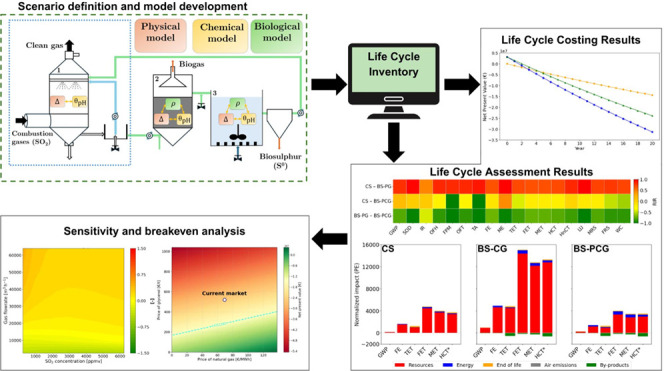

## 1 Introduction

Anthropogenic sulfur dioxide (SO_2_) emissions, primarily derived from fossil fuel combustion, continue to pose substantial risks to ecosystems, public health, and industrial infrastructure.^1–3^ To mitigate these impacts, a variety of flue-gas desulfurization (FGD) approaches have emerged, each designed to capture or convert sulfur compounds under specific technical and economic conditions. Among established technologies, wet scrubbing remains prominent due to its high SO_2_ removal efficiencies.^1^ In parallel, semidry techniques such as spray-dry scrubbers have gained attention for their comparable performance coupled with reduced wastewater generation, making them attractive for retrofits in aging plants.^3,4^

Beyond conventional, mature FGD methods, recent research has focused on adsorptive desulfurization using materials like metal–organic frameworks and tailored zeolites, which offer reduced sorbent requirements alongside potential resource recovery.^5^ Concurrent advances in sulfur chemistry have facilitated the simultaneous removal of multiple contaminants,^6^ while novel acid-gas pretreatment strategies enable enhanced process optimization.^2^ Bioscrubbing, an approach relying on microbial consortia of the sulfur cycle, has garnered substantial interest as part of the circular bioeconomy. In bioscrubbers, SOx is first absorbed as sulfate in an absorption unit. In sequential bioreactors, bacteria metabolize sulfate to sulfide and sulfide to elemental sulfur, providing self-regeneration of the absorbent, reduced chemicals consumption, minimal secondary waste, and continuous sulfur recovery—all in alignment with strict environmental standards.^7,8^ Notably, these bioprocesses can yield valuable byproducts, including biogas.

In tandem with technological innovation, the rise of the circular economy paradigm underscores the importance of resource recovery and life cycle considerations.^9–11^ Rather than regarding sulfur capture simply as a disposal-oriented endeavor, current perspectives advocate for strategies that minimize new extraction and valorize byproducts.^12^ Sulfur mining can disrupt local geochemistry^13^ and degrade air quality,^14^ but circular approaches mitigate these effects by incorporating secondary ores, facilitating material recirculation, and lessening reliance on virgin sources.^15,16^ Market forces and regulatory policies further propel near-zero-waste solutions, thereby reducing both localized environmental burdens and broader climate impacts.^17–19^

Life Cycle Assessment (LCA) has been widely employed to evaluate the environmental trade-offs of desulfurization technologies, including chemical scrubbers, bioscrubbers, and marine exhaust gas cleaning systems. Studies have compared biological and physicochemical approaches for biogas treatment,^20^ assessed scrubber systems in maritime transport,^21,22^ and explored air pollution control in livestock farming.^23^ These analyses highlight that while scrubbers and bioscrubbers reduce emissions of SOx and particulate matter, they may also increase impacts related to energy, infrastructure, and reagents. Despite these advances, no single FGD technology universally satisfies all environmental, technical, and economic constraints.^3^ Consequently, emerging trends point to hybrid solutions combining adsorptive, catalytic, and biological processes to optimize SO_2_ capture while aligning with sustainability goals. Embedding these approaches within broader circular strategies enhances both emission control and long-term environmental performance.

The SONOVA—Sulfur Oxide, Nitrogen Oxide VAlorization—process proposed by Mora et al. merges bioeconomy principles with SO_*x*_-rich flue gas treatment to enable biosulfur recovery. This two-stage bioscrubber first captures SO_2_ as sulfite/sulfate in a slightly alkaline solution and then employs a two-step biological process to convert sulfite/sulfate into sulfide in a UASB reactor, followed by partial oxidation to elemental sulfur in a Continuous Stirred Tank Reactor (CSTR). Initial assessments reported the technical and economical feasibility of integrating physical absorption and biological treatment units using crude glycerol as carbon source for sulfate reduction.^24^

Mathematical models have proven to be valuable tools for optimizing gas treatment processes.^25–27^ Significant efforts have been made to model biofilters and biotrickling filters for sulfur removal, especially in the field of biogas upgrading.^28,29^ These models typically focus on representing mass-transfer phenomena between liquid, solid, and gas phases as well as biofilm growth through biological process rates, with the ultimate goal of predicting the outlet concentrations of target compounds in the gas and/or liquid phase. In contrast, fewer studies have explored full-scale modeling applications for bioscrubbers.^30,31^ Although these models effectively capture the biological degradation of absorbed compounds, they do not consider crucial parameters for economic feasibility, such as chemical demand or operational costs. Moreover, the large-scale implementation of bioscrubbers may be hindered by environmental concerns, including energy requirements, atmospheric emissions, and wastewater management. While previous studies have addressed the development of a mathematical model for the SONOVA bioscrubber, these efforts have focused solely on the UASB bioreactor^32^ and the aerobic oxidation of sulfide.^33^ Consequently, mathematical models for the other key stages of the process—namely, the chemical absorption of SO_2_ and aerobic removal of COD—remain dismissed. Additionally, the integration of algebraic equations is necessary to incorporate essential process control elements, including pH, dissolved oxygen (DO), and temperature control, within the biological stages for a proper estimation of operating costs of the technologies.

This study evaluates for the first time the technical, environmental, and economic feasibility of a SONOVA bioscrubber compared to that of a chemical scrubber to treat sulfur-rich off-gas from an inland pigment facility that combusts elemental sulfur and additives. Our primary advance is an end-to-end, plant-level dynamic model of the full treatment train—gas absorption, sulfidogenic UASB, and microaerobic sulfide oxidation—where physical mass-transfer, chemical speciation, and biological kinetics are explicitly coupled and augmented with process-control equations (pH and DO set-points) and energy-integration correlations. We benchmark this integrated framework against a conventional chemical scrubber (CS) and two bioscrubber variants (BS-PG with fossil glycerin; BS-PCG with purified crude glycerol) that supply distinct carbon substrates to sulfate-reducing microorganisms. By tightly coupling the process model with LCA and Life Cycle Costing (LCC), we generate model-based LCIs and perform dynamic, scenario-resolved evaluations that quantify how purge fraction, gas flow rate, and sulfur load—and their correlations—shape environmental and economic outcomes. We further deliver a model-based breakeven analysis mapping the natural-gas and glycerol price space to identify thresholds at which BS-PCG becomes competitive with CS. In sum, this work contributes to (i) a comprehensive mathematical model accounting for physical, chemical, and biological processes spanning all SONOVA stages; (ii) a multireactor, plant-wide simulation framework that links absorber–UASB–oxidizer units with embedded control logic and energy recovery; and (iii) a fully integrated model–LCA–LCC platform enabling dynamic sensitivity/correlation analyses and quantitative breakeven thresholds to support technology selection under realistic operating conditions.

## 2 Materials and Methods

### 2.1 Goals and Scope of the Study

The study quantitatively compared the traditional chemical scrubber (CS) with the bioscrubber under two distinct carbon-source configurations (BS-PG and BS-PCG) for the abatement of sulfur-rich off-gas from a sulfur-pigment production line, with the recovery as the byproduct of sulfur and biogas for the exploitation in the sulfur combustion chamber. The goals are to (i) assess technical performance and material/energy demands across the full treatment train; (ii) quantify environmental impacts (LCA) and costs (LCC) using model-driven life-cycle inventories; and (iii) identify operating and market conditions under which bioscrubbing is environmentally and economically preferable to CS via dynamic sensitivity, correlation, and breakeven analyses. The functional unit of the system was the annual off-gas volume produced by the combustion chamber and treated by the treatment trains, considering a continuous flow rate of 32,000 m^3^ h^–1^. Three scenarios were assessed in this analysis: CS, BS-PG, with glycerin as carbon source, and BS-PCG with purified glycerol as C source. System boundaries, mass and energy flows, and the specific processes for each scenario are depicted in Figure 1.

**Figure 1:**
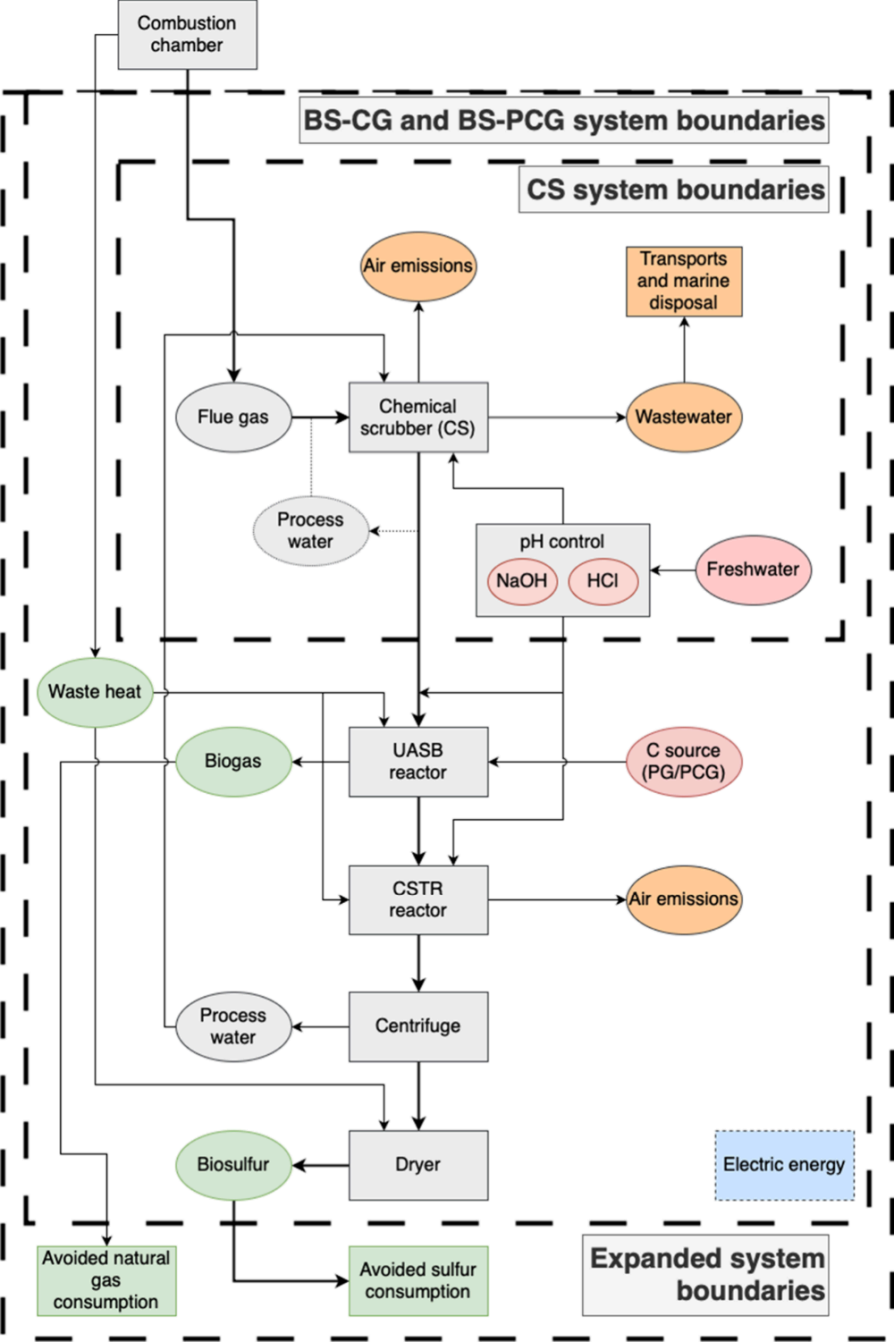
System boundaries of the proposed scenarios. Inner dashed line shows the chemical scrubbing (CS) boundary, the intermediate dashed lines delineate the bioscrubber-crude glycerol (BS-CG) and bioscrubber-purified crude glycerol (BS-PCG) boundaries, and the outer dashed outline marks the expanded system. Byproducts are shaded green, resources red, and end-of-life flows orange; electric energy inputs (blue) apply across all systems (see Table 2 for details). The process water arrow in CS is dotted to indicate recirculation only for this scenario, whereas dewatering-water recirculation is active in the BS-CG and BS-PCG scenarios.

In CS, conventional chemical scrubbers are employed where water circulates in a closed loop with only a minor purge flow. Freshwater quality is based on conditions at the plant’s location, and the off-gas stream originates from the pigment production process. BS-PG introduces a bioscrubber placed downstream of the absorption tower using two reactors to recover sulfur and generate biogas. Finally, BS-PCG replicates the bioscrubber setup but replaces the fossil-based glycerin in the UASB reactor with purified crude glycerol (PCG)—a byproduct from biorefinery processes—while retaining the same overall recovery scheme. The byproducts obtained are directly introduced into the combustion chamber for pigment production, and the analysis explores whether reintegrating these outputs into the process yields lower overall impacts compared to the conventional chemical scrubber. In all scenarios, the only end-of-life waste stream is purged wastewater (brine). This stream is modeled using the corresponding Ecoinvent processes as transport to marine disposal and as direct emissions of nitrogen and phosphorus to seawater. Other brine constituents (e.g., heavy metals, additional ions, and pH-related effects) are not explicitly inventoried, as their concentrations—and thus their ecotoxicity profiles—are expected to be comparable across the three scenarios given the similar production processes and amount.

### 2.2 System Boundaries and Scenarios Investigated

The LCA was conducted in accordance with ISO 14044,^34^ covering gate-to-grave boundaries that begin at the off-gas exiting the combustion chamber and extend through the end-of-life stage. Byproducts were modeled through system expansion and treated as avoided impacts linked to resource use.^35^ They are fed back into the production line by employing biogas as a fuel and sulfur as a raw material in the combustion chamber for pigment manufacturing, based on earlier findings that the same final product output is preserved. This approach captures the environmental benefits of resource recovery while maintaining product consistency throughout the life cycle.

BS-PG was modeled with the glycerin-specific Ecoinvent process, while for BS-PCG, a vacuum distillation process was chosen as it uses fewer resources and has a purification efficiency that allows the final product to be compared to the technical grade (>95%), making it suitable for the process. The impacts related to the purification process were defined through the inventory outlined by.^36^

In this study, the upstream burdens of crude glycerol production were excluded, consistently with a cutoff approach that treats crude glycerol as a minor coproduct of biodiesel with no allocated environmental load.^37^ By contrast, all chemical and energy inputs required for vacuum distillation were fully (100%) assigned to the purified glycerol since this transformation serves only that specific output. Transport of PCG was also included: for consistency, the same transport distances and modes used in the Ecoinvent process for PG were applied to PCG. To allocate the remaining emissions, a mass-based (biophysical) criterion was selected, ensuring methodological consistency with the physical ratio between crude glycerol and biodiesel and avoiding market volatility. Waste streams, including distillation residues, were consequently allocated using a 1:10 ratio reflecting the biophysical proportion of glycerol to biodiesel. This analysis was carried out considering a set of baseline conditions of the scrubbing process, which are summarized in Table 1. These values were defined according to the operating conditions of the pigment production plant, and they were used as inputs for the developed CS/BS mathematical model (see Figure 1). The remaining relevant operating parameters utilized in the study can be found in the Supporting Information (Table S1).

**Table 1:** Operating Conditions of the Chemical Scrubber Utilized to Treat the Combustion Gases of the Pigment Production Plant

variable	units	baseline value
Combustion gas flow rate	[m^3^ h^–1^]	32,000
SO_2_ concentration in the gas	ppm_v_	3,120
CO_2_ concentration in the gas	ppm_v_	3,200
G/L ratio in the absorber	[m^3^_gas_ m^–3^_liq_]	145.5
Purge percentage	[%]	1
Distance from the facility to sea	K m	50

All scenarios were modeled as described in the following section. The model outcomes used as Life Cycle Inventory (LCI) and the Ecoinvent process selected are reported in Tables 2 and S2 (Supporting Information).

**Table 2:** Process Performance of the Three Scenarios—CS, BS-PG, and BS-PCG—with the Gas Influent Conditions of the Pigment Production Plant (See Tables 1 and S1)

**process performance KPI**	**CS**	**BS-PG**	**BS-PCG**
SO_2_ absorption efficiency (%)	59.3	59.9	59.9
SO_4_^2–^/SO_3_^2–^ concentration in the scrubber liquid effluent (g L^–1^)	26.8	0.3	0.3
SO_2_ concentration in the scrubber gas effluent (ppm_v_)	1,186.6	1,167.4	1,167.4
CO_2_ concentration in the scrubber gas effluent (ppm_v_)	2,990.8	5,620	5,620
Energy recovery (%)	0	71.1	71.1
Life Cycle Inventory			
Absorber volume (m^3^)	3.70	3.70	3.70
CSTR volume (m^3^)	0.0	2277.0	2277.0
UASB volume (m^3^)	0.0	1000.3	1000.3
Recirculation flow rate (m^3^ h^–1^)	220.0	220.0	220.0
Caustic flow rate (m^3^ h^–1^)	0.26	0.28	0.28
Discharge flow rate (m^3^ h^–1^)	2.20	2.20	2.20
Freshwater flow rate (m^3^ h^–1^)	2.71	2.81	2.81
Centrifuge feeding flow rate (m^3^ h^–1^)	0.0	220.0	220.0
Dryer inlet flow rate (m^3^ h^–1^)	0.00	18.79	18.79
Dryer blower flow rate (m^3^ h^–1^)	0.00	5.64	5.64
Aeration blower flow rate (m^3^ h^–1^)	0.0	100.0	100.0
Reactors heating pump flow rate (m^3^ h^–1^)	0.0	216.5	216.5
Total freshwater (m^3^ year^–1^)	23,757	24,661	24,661
NaOH 100% (t year^–1^)	1,160	1,231	1,231
Glycerine (t year^–1^)	0.0	4,094.6	0.0
Purified Glycerol (t year^–1^)	0.0	0.0	4,310.1
Recirculation pump (KWh year^–1^)	156,210	0	0
Caustic pump (KWh year^–1^)	144.96	153.84	153.84
Discharge pump (KWh year^–1^)	1,783.9	1,783.9	1,783.9
Freshwater pump (KWh year^–1^)	1,484.8	1,541.3	1,541.3
Carbon source pump (KWh year^–1^)	0.00	0.57	0.60
Centrifuge feeding pump (KWh year^–1^)	0	156,210	156,210
Dryer inlet pump (KWh year^–1^)	0	10,296	10,296
Dryer blower pump (KWh year^–1^)	0.0	2,471.2	2,471.2
Aeration blower pump (KWh year^–1^)	0	29,220	29,220
Reactors heating pump (KWh year^–1^)	0	153,748	153,748
Centrifuge (KWh year^–1^)	0.00	2.18	2.18
Medium to low power transformation (KWh year^–1^)	159,624	355,427	355,427
Wastewater (KWh year^–1^)	19,285	19,272	19,272
Transports (t km)	964,260	963,600	963,600
CO_2_ (CSTR) (kg year^–1^)	0	637,767	637,767
SO_2_ (CSTR) (kg year^–1^)	0.00	2.18	2.18
CO_2_ (flashlight) (kg year^–1^)	0	154,712	154,712
Biogas (m^3^ year^–1^)	0	–699,871	–699,871
Sulfur (t year^–1^)	0.00	–356.92	–356.92

### 2.3 Mathematical Model Description

The mathematical model for all three scenarios was developed by using MATLAB R2023a. Each stage of the process is governed by a set of differential equations, which were solved using the ode15s solver. As shown in Figure 1, the bioscrubber consist of an absorber followed by a sequential anaerobic–aerobic biological treatment. After SOx absorption, the UASB bioreactor is fed with glycerin or PCG which serves as a carbon and electron source for bacterial growth and sulfate reduction. Second, the CSTR is aerated to ensure sulfide oxidation. The process culminates in the recovery of biosulfur (S^0^_b_) as a solid product through centrifugation and sulfur drying, enabling its potential reuse.

The model integrates physical and biological processes across the different stages, which can be considered as submodels that dictate the temporal dynamics of the state variables. Also, the inclusion of a pH model is fundamental to the aims of this study because (1) it allows for the proper calculation of the mass-transfer rates between liquid and gas phases for ionic species—such as inorganic carbon, sulfite, and sulfide—and (2) it enables one to accurately quantify the amount of chemicals—acid as HCl or base as NaOH—added to control the pH within a desired range, which is necessary to optimize biological activity. A detailed description of the pH model and the corresponding algebraic equations is provided in the Supporting Information (SM1.1). This section aims to summarize the main modeling assumptions and the application of these submodels to the different stages of the bioscrubber—scrubber, UASB, and CSTR.

#### 2.3.1 Chemical Absorber

The absorber model was formulated as a series of continuously stirred tank reactors (CSTRs), in which mass transfer occurs between the gas and liquid phases. The model was calibrated and validated using experimental data from a lab-scale spray scrubber.^38^ The integration of a mass-transfer coefficient (*k*_L_) correlation^39^ as well as the model calibration and validation results are provided in the Supporting Information (see SM1.2).

#### 2.3.2 UASB Bioreactor for Sulfate Reduction

The sulfidogenic UASB bioreactor fed with glycerin or PCG had been previously modeled.^32^ This model integrates chemical, biological, and physical processes. Calibration and validation were performed using independent experimental runs of a lab-scale UASB reactor operated with crude glycerol and sulfate. The model is based on the following key assumptions:

1. Hydraulically, the system is modeled as a series of mini-CSTRs to capture the plug-flow-like behavior of granular biomass within the reactor. This representation was considered essential for accurately simulating the inhibition effects caused by the accumulation of impurities throughout the reactor.
2. The biochemical model consists of 15 biological reactions carried out by 3 different trophic groups: fermentation (8), sulfate-reduction (5), and methanogenic (2) processes. Growth kinetics were described using Monod-type equations incorporating inhibition terms for sulfide and crude glycerol impurities. However, since crude glycerol was not utilized in the considered scenarios, its associated inhibition function was omitted.
3. Mass-transfer processes between the liquid and gas phases take place on top of the reactor assuming an overpressure in the headspace. The mass-transfer coefficient (*k*_L_ *a*) was assumed as 200 day^–1^.^40^

The utilized model had been proven to predict the outcome of the main C and S species both in the liquid and gas phases^32^ and thus was considered suitable for use in this study.

#### 2.3.3 Aerated Mixed Tank Bioreactor for Sulfide Oxidation

To incorporate the kinetics of aerobic sulfide oxidation, the biochemical model proposed by Mora et al.^41^ was integrated into this study. This model accounts for the growth kinetics of sulfur-oxidizing bacteria (SOB), which utilize sulfide, thiosulfate, and elemental sulfur as electron donors. Additionally, the kinetics of facultative heterotrophic biomass were adapted from the Activated Sludge Model No. 2,^42^ considering that the system may receive organic carbon depending on the performance of the upstream UASB bioreactor.

A dissolved oxygen (DO) set point of 0.1 mgO_2_/L was maintained using a simulated proportional-integral (PI) controller (see SM1.3) to enhance partial sulfide oxidation. The controlled variable was the air flow rate supplied, and a correlation to calculate the volumetric mass-transfer coefficient (*k*_La_) by Pittoors et al.^43^ was utilized to describe the DO fluctuations in the liquid phase. All of the relevant information regarding the bioprocess reactions and degradation pathways as well as the control systems for pH and DO is included in the Supporting Information (SM1.1 and SM1.3).

#### 2.3.4 Energy Utilization

Finally, the temperature was not included as a state variable in the model, but some assumptions were made to calculate the percentage of utilized available energy coming from the hot combustion gases:

1. The combustion gases exit the furnace at 400 °C and enter the absorption stage at 80 °C according to data provided by the pigment production facility.
2. The liquid effluent from the absorber has an average temperature of 25 °C throughout the year, while the liquid influent to the biological stages must be heated to 35 °C as this is the operating temperature of the biological units.
3. The sludge from the CSTR contains 65% of humidity after centrifugation and must be dried down to 10% for S^0^_b_ recovery. The dryer uses air at 140 °C.
4. Two heat exchangers are utilized: (1) an air/air heat exchanger for sludge drying and (2) an air/water heat exchanger to raise the jacket water temperature for the biological stages from 25 to 35 °C.

The corresponding equations to calculate the percentage of utilized energy and pump and centrifuge energy consumption are provided in the Supporting Information (SM1.4)

### 2.4 Life Cycle Impact Assessment (LCIA)

The LCIA was conducted using the ReCiPe 2016 method, chosen for its extensive range of midpoint indicators and its incorporation of globally applicable impact mechanisms.^44^ The Hierarchist perspective was adopted because it enables the assessment of long-term impacts—a critical aspect of strong LCIA—and is widely recognized in scientific literature.^44,45^

All of the impact categories were evaluated: Global Warming Potential (GWP), Stratospheric Ozone Depletion (SOD), Ionizing Radiation (IR), Ozone Formation for Human Health (OFH), Fine Particulate Matter Formation (FPMF), Ozone Formation for Terrestrial Ecosystems (OFT), Terrestrial Acidification (TA), Freshwater Eutrophication (FE), Marine Eutrophication (ME), Terrestrial Ecotoxicity (TET), Freshwater Ecotoxicity (FET), Marine Ecotoxicity (MET), Human Carcinogenic Toxicity (HCT), Human Non-Carcinogenic Toxicity (HNCT), Land Use (LU), Mineral Resource Scarcity (MRS), Fossil Resource Scarcity (FRS), and Water Consumption (WC). Based on the LCIA results, the relative impact ratio (RIR) was calculated as

$$\mathrm{RIR}=\frac{{\mathrm{IC}}_{\mathrm{BS}2}-{\mathrm{IC}}_{\mathrm{CS}}}{\max({\mathrm{IC}}_{\mathrm{BS}2},{\mathrm{IC}}_{\mathrm{CS}})}$$ 1

A heatmap was constructed to facilitate pairwise comparisons between the scenarios for each impact category. In this visualization, green areas (RIR < −0.5) denote a higher impact for the CS scenario, yellow areas (−0.5 < RIR < 0.5) indicate comparable impacts between the scenarios, and red areas (RIR > 0.5) signify a higher impact for the BS-PCG scenario.

Normalization was performed using ReCiPe normalization scores, which express impacts in person-equivalents (PE)—a metric representing the average annual impact of a single individual’s activities needed to sustain the current standard of living.^46^

The normalized results were used to rank the impact categories, identify the most significant impact categories (top 5 and GWP, representing more than 95% of total impact), and perform a contribution analysis. Based on that, sensitivity and break-even analyses were further carried out to explore how variations in plant configuration and location influence the results.

### 2.5 Life Cycle Costing

Similarly to the LCA perspective, LCC has the purpose of assessing a system or a product, from an economic and financial perspective, during its life span. For this case study, a lifetime of 20 years was assumed for the LCC analysis.

The LCC analysis encompassed investment costs, operating expenses, labor costs, wastewater disposal costs, and revenues from byproducts. A market survey was conducted to estimate operating expenses for each scenario, allowing for the identification of both fixed and variable cost components—where variable costs depend on the flow rate—thereby yielding final costs that vary according to the model. Certain cost items, such as the demister and electrical substation, were considered fixed because they do not scale with plant size.

Operating costs were derived from literature data or official pricing information for raw materials on the European market, referred to 2021 and representative of an economically stable situation. The cost of NaOH, based on a commercial quotation for continuous supply, was determined to be €527 per ton. Pure glycerin was priced at €745 per ton, whereas purified glycerol was valued at €520 per ton. Sulfur cost was set at €150 per ton. Electricity and natural gas costs were taken as the European Union averages, at €0.088/kWh and €0.354 per m^3^, respectively. The cost for freshwater procurement and wastewater transport corresponded to the actual amounts paid by the company at €0.06 per m^3^ and €7.23 per m^3^, respectively. Labor costs were calculated using average European wages for each professional category, assuming a 37.5 h work week and a tax wedge of 35%. Maintenance costs were estimated as a percentage of the investment costs, allocating 2% to the chemical scrubber, 5% to the other reactors and major components, and 10% to all remaining components. The complete list of relative costs used for LCC and their estimation year is shown in Tables S3 and S4 of the Supporting Information.

LCC was performed adopting the same functional unit (FU) as the LCA. To assess the cost-effectiveness of each scenario, both the Net Present Value (NPV) and the Internal Rate of Return (IRR) were computed.^47^ NPV estimates the current value of a series of future cash flows from an investment, calculated by subtracting the present value of outgoing cash flows (expenses, including investment costs) from the present value of incoming cash flows (revenues), as expressed in eq 2:

$$\mathrm{NPV}=\sum_{t=1}^{N}\left[\frac{R_{t}}{(1+i{)}^{t}}\right]$$ 2

In this study, *N* represents the total number of years considered (20 years), *t* denotes each time period (1 year, consistent with the adopted functional unit), *R_t_* is the cash flow in period *t*, and *i* is the interest rate.

An interest rate of 1.5% was adopted for the present analysis. This value is consistent with long-term real rates recommended by NIST for capital budgeting in stable financial environments^48^ and lies at the lower bound of the 1–3% social discount rate range increasingly advocated for long-term public, climate, and sustainability investments^49^ in coherence with the Eurocontrol’s guidance for intergenerational or environmental projects.^50^ The internal rate of return (IRR)—defined as the rate at which the NPV equals zero—is used to measure investment profitability. However, since the analysis focuses on the gas treatment line, excluding pigment production, both the cash flow and the NPV are negative, rendering the IRR less meaningful. Consequently, rather than performing a sensitivity analysis based on the traditional NPV variation with discount rates, we adopted the approach described by ref 51 to calculate the cumulative change in present value. Furthermore, the cumulative present value was employed as the basis for the sensitivity analysis on discount rates because, in the context of negative cash flows, the traditional NPV proves to be uninformative. This methodological choice enables a more reliable assessment of the impact of variations in the interest rate on economic analysis.

### 2.6 Sensitivity and Breakeven Analysis

The sensitivity and breakeven analysis was conducted by simultaneously varying key process parameters: gas flow rate (m^3^ h^–1^), inlet SO_2_ concentration (ppmv), water purge fraction (%), and distance to the sea (km). For each simulated scenario, the top 5 LCA impact categories, GW, operational costs, and investment costs were computed, and the RIR was calculated as previously defined in eq 1. Contour plots were generated to compare the CS and BS-PCG scenarios, while supplementary figures (Figures S5 and S6) detailed variations in reagent consumption under different operating conditions, thereby identifying conditions where BS-PCG outperforms CS.

## 3 Results and Discussion

### 3.1 Baseline Process Performance Comparison

Table 2 shows the main process performance results and the complete LCI of the three scenarios analyzed in this study.

These results portray the advantages and limitations of the bioscrubber in both scenarios—BS-PG and BS-PCG—with respect to CS. On one hand, process performance improves with the integration of bioprocesses, particularly in terms of resource recovery and the production of value-added byproducts—methane and elemental sulfur. As a result, effluent quality is improved in terms of S compounds concentration, since a considerable amount—about 54%—of the treated S is recovered in the form of elemental sulfur. This recovered sulfur can then be reintegrated as sulfur-rich biomass into the pigment production line directly in the combustion chamber without further pretreatment after the drying phase. Moreover, the implementation of a heat recovery system for the bioprocess units enables the use of energy (71%) otherwise lost during the combustion process.

However, a key drawback of incorporating bioprocesses is the increased demand for chemicals. A big part of it is the need for carbon sources to reduce sulfate in the UASB bioreactor. This fact could potentially harm the environmental and economic feasibility of the process due to transportation costs of the C source and the emission of CO_2_ in both biological units.^52,53^ In this context, the use of PCG (BS-PCG scenario) presents a promising alternative, as it is a low-cost byproduct of the biodiesel industry and widely available in the market.^54,55^ Nonetheless, in terms of caustic consumption, the removal of acidic sulfur species—sulfate, sulfite, and sulfide—within the biological units helps mitigate the acidification of the liquid stream in the scrubber. As a result, the demand for NaOH in the bioscrubber scenarios is reduced by 46%.

### 3.2 LCA Outcomes

With the results of the mathematical model, an LCIA analysis was conducted for the three identified scenarios, and the results are reported in Table S5, where the negative values indicate that the avoided impacts are greater than the direct impacts (positive values) generated by the systems, based on previous assumptions. The comparison between BS-PG and BS-PCG to CS and between themselves as potential environmental impacts percentage differences are depicted in the heatmap in Figure 2A. Better performance of the second term is reported in green, comparable performance in yellow, and worst performance in red. Figure 2B–D report the contribution analysis of the top 6 impactful categories.

**Figure 2:**
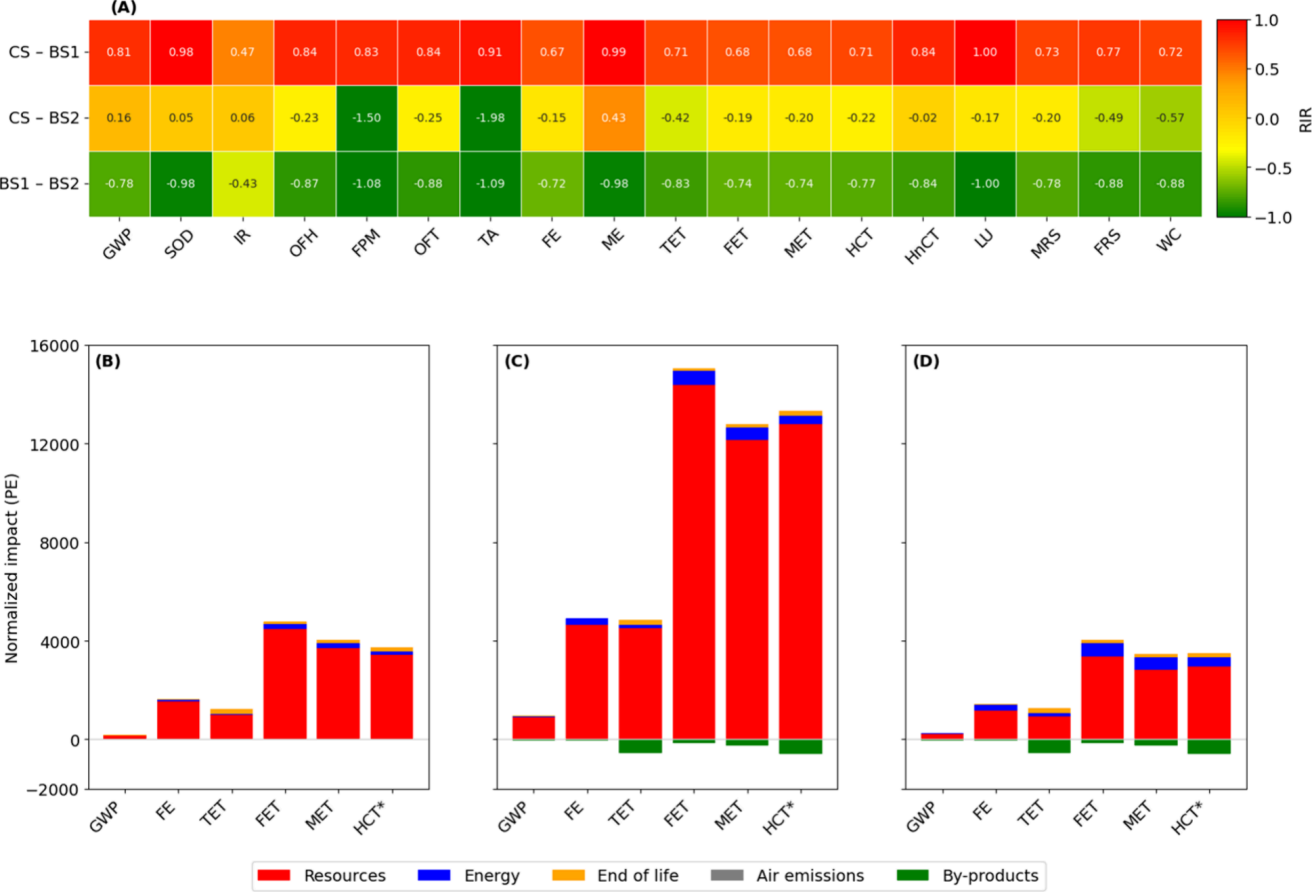
Comparative environmental assessment of scenarios through Relative Impact Ratio (RIR) and contribution analysis. (A) Heatmap of the RIR values for the impact categories across the three scenarios: CS, BS-PG, and BS-PCG. (B–D) Normalized environmental impacts for the most relevant categories of CS (B), BS-PG (C), and BS-PCG (D), highlighting the contribution of individual impact components: Resources (red), Energy (blue), End of life (orange), Air emissions (gray), and Byproducts (green). Note: The value for HCT (Human Carcinogenic Toxicity) has been divided by a factor of 10 to improve the readability.

The comparison of scenarios shows that BS-PG loses to both CS and BS-PCG, performing worse in each category except for IR which is comparable between CS and BS-PG. In contrast, the comparison between CS and BS-PCG is more balanced, with 4 categories where BS-PCG performs better (Δ > 0.5), 13 categories where results are comparable (−0.5 > Δ > 0.5), and only 1 category where BS-PCG performs worse (Δ < −0.5).

BS-PCG’s have the worst performance only for ME, due to the PCF footprint characterized by HCl consumption and wastewater production. In contrast, the categories in which BS-PCG performs better are FPM, TA, FRS, and WC. For the first two, the cause is the impacts avoided by the recovery of sulfur as a raw material realized in GLO, which is not appreciable in GLY due to the high impact of using pure glycerin as a carbon source. For WC, on the other hand, the main role is that of purified glycerol, due to 0.11 m^3^ of wastewater to be treated per kg of purified glycerol produced by vacuum distillation and responsible of avoided impacts.^36^

As carried out in a previous work,^56^ total cumulative normalized impacts were calculated. CS is characterized by an impact of 51,090 PE, BS-PG 178,623 PE, and BS-PCG 39,692 PE. The normalized values for each category of each scenario are reported within the Supporting Information (Tabel S7). Based on these results, a percentage contribution was attributed to each category and, based on the reference scenario, the 5 most impactful categories (HCT, FET, MET, FE, TET) were selected, including also the GWP due to its relevance in scientific and policy contexts.^57–59^ The results and corresponding contribution analyses for the three scenarios evaluated are presented in Figure 2 (B: CS, C: BS-PG, D: BS-PCG). The impact categories shown collectively account for at least 95% of the total environmental impact, ensuring that the comparison is representative for all scenarios assessed.

Even reducing the analysis to only 6 categories, BS-PG was confirmed as the worst-case scenario, while CS and BS-PCG were comparable. In BS-PCG, benefits from byproducts were identified, the impact of which guarantees better performance of BS-PCG than CS. The contribution analysis shows resources as the predominant life cycle step for defining the impacts, linked to the high NaOH consumption for CS and the C source for BS-PG and BS-PCG. The large difference between BS-PG and BS-PCG and the comparability of CS and BS-PCG is therefore determined by the impacts related to the carbon source used. Purified glycerol, being a byproduct coming from an organic source, has a much lower footprint than fossil glycerin, confirming that it is a low-impact alternative C source that can be used in biological processes. The environmental footprint of purified glycerol was mainly determined by the consumption of HCl and heat required for the distillation process (0.12 kg of HCl and 1.07 MJ of heat for steam production). The possible application of thermal waste for steam heating would allow the reduction of impacts related to this resource, making BS-PCG even more competitive as alternative to CS.^60^

The byproduct production for BS-PCG failed to compensate the impacts related to the other life cycle stages, with the exception of FPMF and TA. Besides these two categories, substantial mitigation from byproducts was achieved for GWP, OFH, OFT, TET, HCT, and FRS (−22.9, −40.7, −47.6, −76.8, −21.0, and −217.3%). Biogas recovery, modeled as avoided natural gas consumed, determined the major environmental gains, especially for FRS, confirming the importance of the recovery of this resource in biological processes for the mitigation of impacts as demonstrated in numerous studies.^47,61^ Sulfur recovery, the main focus of the SONOVA process, has a significant contribution (>25% of total avoided impacts) only for SOD, FPMF, TA, TET, and MET. In fact, they are the largest impact items (following normalization) related to sulfur use together with HCT, whose compensation is mostly due to biogas recovery.

The impact deriving from energy consumption is differently modulated for the three scenarios compared. In CS and BS-PG, it has a marginal effect, with a significant impact (>10%) only for the IR category. In BS-PCG instead, due to the reduced impacts related to resources and the presence of the downstream phase compared to CS, the energy contribution was more pronounced, with significant impacts on all categories except ME. The largest contribution was made to IR and FRS (48.1 and 37.0%), while for FET, MET, and HCT (indicated in Figure 2D), the contribution was slightly above 15%. These results confirm the importance of energy sources in defining the impacts of biological processes as already amply demonstrated in literature.^62^

Atmospheric emissions only contributed to the GWP in BS-PG due to the biogas produced by the CSTR and burned in the flashlight for complete CO_2_ conversion. While for BS-PCG atmospheric emissions were related to the emissions of CO_2_ to the atmosphere, impacting FPMF and TA with negligible contribution (<1%), CS produced 1379 tCO_2eq_ entirely from the life cycle and not from plant emissions. BS-PG and BS-PCG, on the other hand, have an output of 7,277 and 1,599 tCO_2eq_, respectively, with direct emissions contributing less than 0.1% for both cases.

The main advantage of the SONOVA process is the reduced amount of NaOH needed to reduce the SO_2_ concentration in the flue gas, with almost halved NaOH consumption that strongly reduces the impacts associated with the consumption of this resource. This fact is a consequence of an improved sulfate and sulfite removal in the biological treatment units. The significant environmental footprint associated with the carbon source, driven by the high HCl and heat consumption during its purification, prevent a clear advantage of BS-PCG over CS. By reducing the impacts related to the purification process or by changing the C source used, the SONOVA process could gain further advantages over the baseline CS, improving the environmental sustainability of the bioscrubbing process.

Finally, the end-of-life step appeared to have a measurable contribution only for CS and BS-PCG, with relevant impacts for TET and LU for the first and OFT, TET, and FRS for the second. This stage represented a key assumption of the study, as the end-of-life for CS was modeled as marine disposal, an area still under methodological development.^63^ Ecotoxicological impacts from brine component emissions were not calculated due to current limitations in LCIA models. However, given the consistent effluent characteristics and production across scenarios, this simplification did not affect the comparative analysis and BS-PCG remained the environmentally preferable option.

### 3.3 LCC Outcomes

In Table 3, the main economic analysis results are reported, while the complete lists of Operative costs, investment costs, maintaining costs, and labor costs are reported in Tables S7–S10 of Supporting Information.

**Table 3:** Summary of the Main Results from Life Cycle Costing (LCC) Analysis for the Three Scenarios: CS, BS-PG, and BS-PCG

indicator	CS	BS-PG	BS-PCG
Investment costs (€)	–51,672	–3,191,321	–3,191,322
Operative costs and revenues (€/year)	–827,067	–1,832,802	–1,399,945
Byproduct earnings (€/year)	0	335,701	335,701
Labor costs (€/year)	–6,395	–28,473	–28,473
Maintaining costs (€/year)	–2,885	–131,699	–131,699
Annual cashflow (€/year)	–836,348	–1,992,974	–1,560,117
NPV (€)	–14,311,282	–31,272,606	–23,841,044
IRR (%)	1,618	–3,191,321	–3,191,322

CS was the most cost-effective choice for each cost item. In fact, revenues from the sale of byproducts did not offset the operating costs of purchasing the carbon source, even in the case of purified glycerol. Maintenance and investment costs were also significantly lower for CS due to the lower complexity of the process and the presence of a single reactor. The operating costs of CS are mainly due to the consumption of NaOH (672,161 €/year) and the disposal of wastewater (139,432 €/year). The distance of the plant from the sea was therefore also a cost factor herein, although to a much lesser extent than the environmental impacts. For BS-PG and BS-PCG, the costs related to NaOH were lower (−356,441 €) and the higher operating costs were due to the consumption of the C source (1,631,720 €/year for BS-PG and −1,198,862 €/year for BS-PCG), which led to a clear increase in operating costs for these two scenarios. The gains from byproducts cannot compensate for this extra cost, leading CS to be consistently the best scenario from an economic point of view. The costs related to HCl consumption for BS-PG and BS-PCG were negligible.

Since the scope of the analysis does not include pigment production but only the off-gases treatment line, cashflow and NPV were negative, leading the IRR to assume a meaningless value. Therefore, instead of the Cumulative NPV as IR changes, which is typical of these studies,^47^ the Cumulative Present Value change was calculated according to ref 51. The results of this analysis (Figure S4) show that, for any interest rate and at any time in the life cycle, CS was the most economically advantageous scenario.

Net of these results, the distance of the plant from the sea and the amount of purged water were key in the comparison of these scenarios, as the former directly affects the impacts related to effluent transport, while the latter affects both the amount of effluent and the amount of NaOH and C source required for the functionality of the plant. Furthermore, based on the specific knowledge of the processes involved and the relevant literature,^64–66^ the flow rate and the sulfur concentration at the scrubber inlet and purged water are key driver parameters of the processes examined. Based on this and the role of distance traveled for marine disposal, the above-mentioned parameters were used for sensitivity and breakeven analysis. Their influence on the main environmental impacts and cost items was studied (see Section 3.4). Due to their nature, two pairs of parameters with common elements were formed for a comprehensive study: (a) purged water and distance and (b) flow rate and S concentration.

For these pairs, it was verified how they influence the comparison between the two scenarios found to be comparable (CS and BS-PCG), through the analysis of the variability of the percentage deviations shown through the heatmap in Figure 2. The results obtained were plotted on 2D graphs, reported in Figures 3 and 4. Finally, considering the importance of the costs of the C source and natural gas, a breakeven analysis was carried out that relates these two cost variables, identifying an area within which BS-PCG is economically competitive. The results of that last analysis are reported in Figure 5.

**Figure 3:**
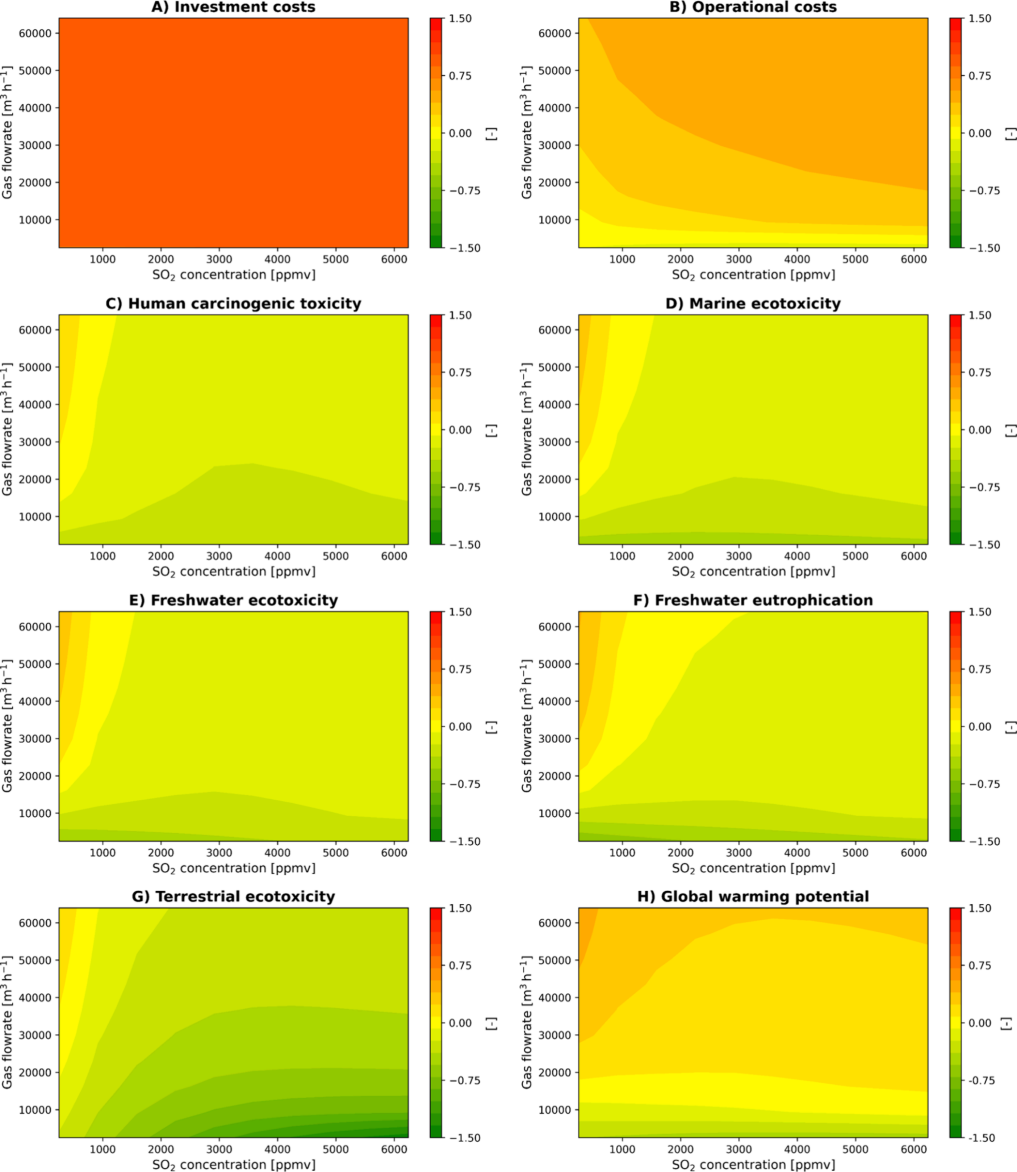
Impact of gas flow rate and SO_2_ concentration on CS and BS-PCG scenarios across eight key LCA and LCC KPIs.

**Figure 4:**
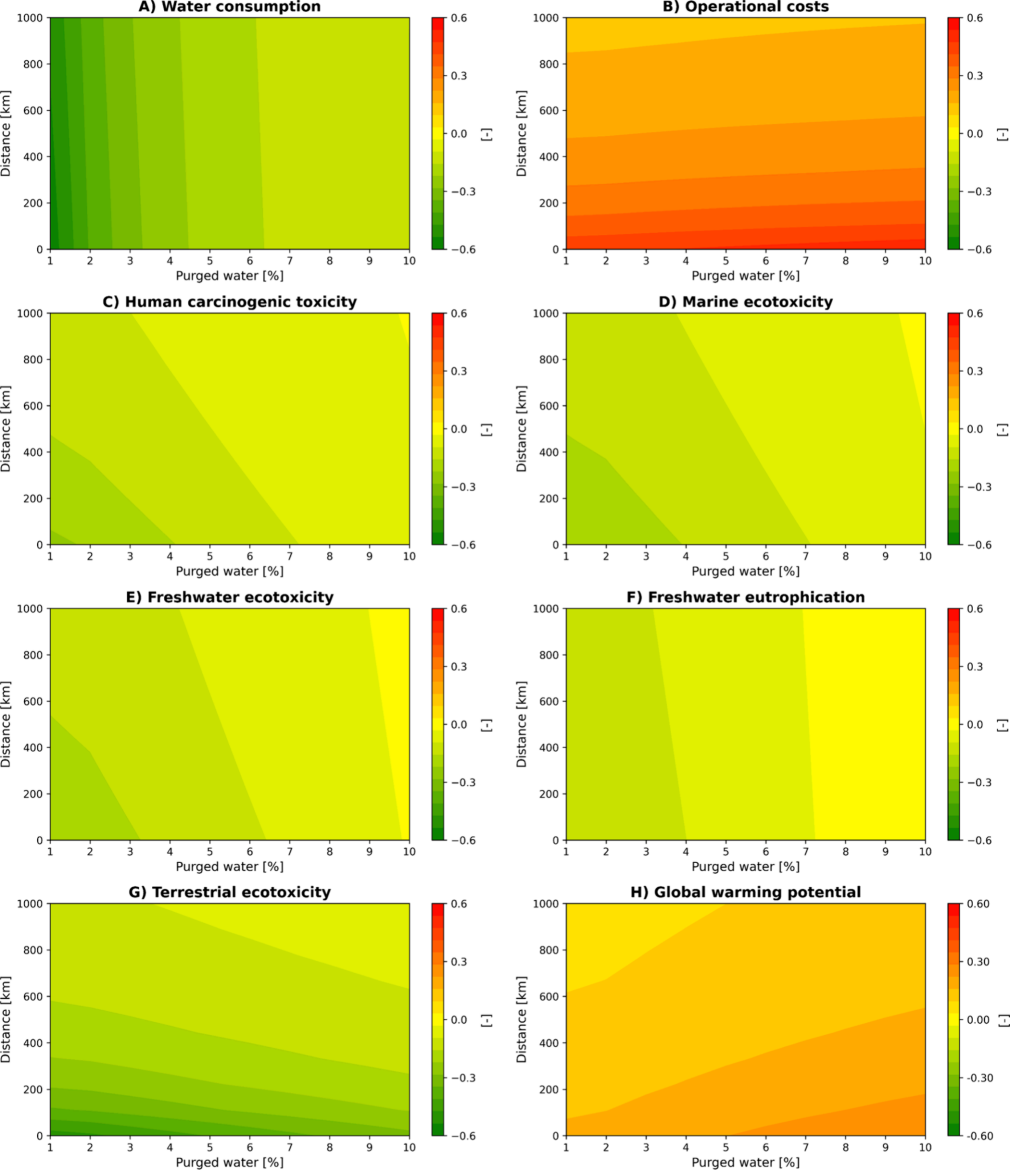
Impact of purged water and distance of plant to sea on CS and BS-PCG scenarios across eight key LCA and LCC KPIs.

**Figure 5:**
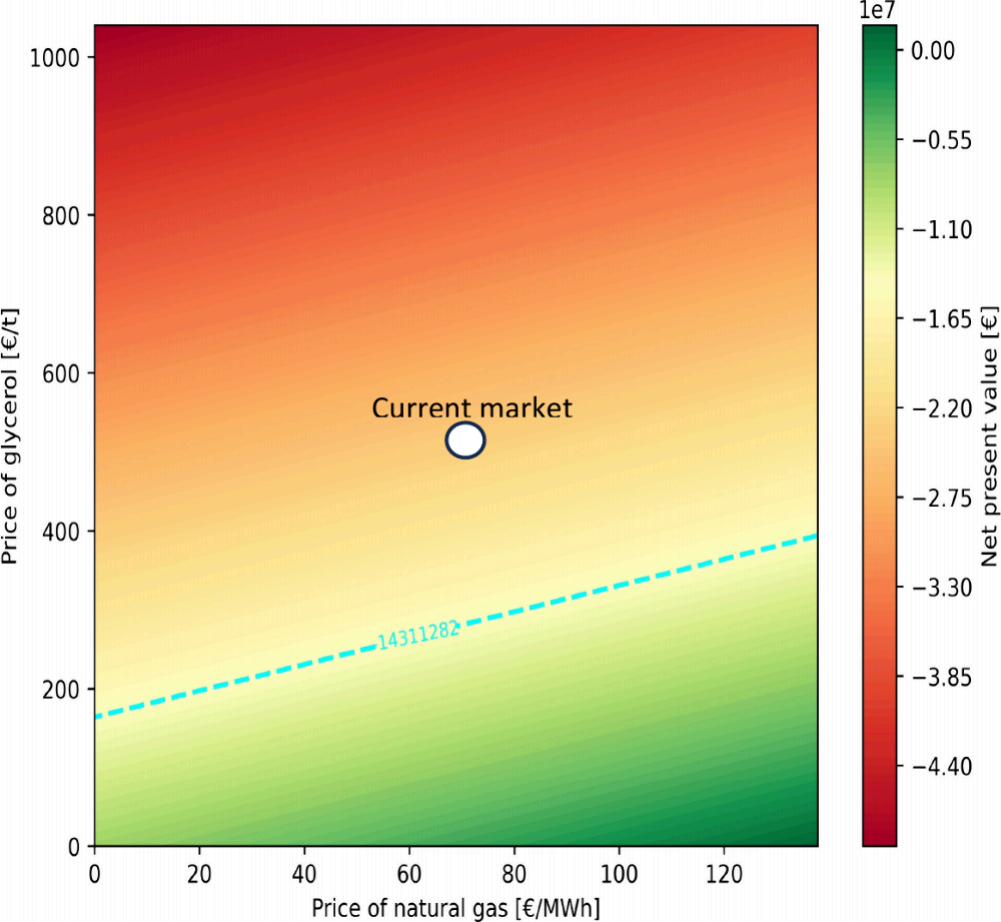
Breakeven analysis of BS-PCG economic feasibility, represented by the net present value [€], as a function of natural gas and purified crude glycerol prices. The dashed line represents the conditions in which the NPV of BS2 is equal to that of CS—with a value of −14,311,282 €. The white circle represents the current market conditions, where the price of glycerol is 520 €/Tn and the price of natural gas is 68.9 €/MWh.

### 3.4 Sensitivity and Breakeven Analyses Using the Most Relevant LCA-LCC KPIs

Figure 3 comprises eight contour plots each illustrating how the CS compares with BS-PCG as the gas flow rate (vertical axis) and inlet SO_2_ concentration (horizontal axis) increase. The color scale transitions from green to red, where green indicates that BS-PCG achieves lower costs or environmental impacts, while red signifies that CS outperforms BS-PCG in the corresponding category.

To better contextualize these observations, Figure S5 in the Supporting Information provides essential insight by illustrating variations in caustic and WC within CS and BS-PCG, as well as crude glycerol consumption and sulfur recovery in BS-PCG, under the analyzed conditions. In Figure S5A, it is evident that caustic consumption in BS-PCG has a higher rise than that in CS with increased SO_2_ concentrations and gas flow rates. This trend highlights intensified requirements for pH control in the biological units at larger operational scales or under conditions of elevated sulfur load in the inlet gas. Consequently, precise pH control becomes more critical, resulting in greater caustic use. Analogously, crude glycerol consumption in BS-PCG also intensifies alongside rising inlet SO_2_ concentrations and flow rates (Figure S5C), underscoring that increased sulfur loading requires a corresponding increase of carbon source input to adequately sustain sulfate reduction in the UASB.

In Figure 3, both investment costs (A) and operational costs (B) remain predominantly red and orange, respectively, reflecting that CS consistently requires lower capital outlays and day-to-day expenditures, even at larger scales or elevated sulfur loads. The additional reactors and control systems in BS-PCG, along with the carbon source requirement, do not yield sufficient economic benefits to offset their higher complexity.

We can also observe that HCT (C) is predominantly green but shifts toward orange at higher flow rates and low SO_2_ concentrations, suggesting that BS-PCG gains some advantage in lower-sized plants scenarios. These results are aligned to the trends identified for MET (D), FET (E), and freshwater eutrophication (F). This pattern points to a mitigation of aquatic impacts in BS-PCG at higher throughputs. TE (G) becomes more favorable to BS-PCG when the system operates at smaller scales and high sulfur concentrations, whereas GWP (H) remains firmly in favor of CS throughout the evaluated range, with the exception of small-sized plants – below 10,000 m^3^ h^–1^ of treated gas.

BS-PCG gains competitivity at low gas flow rates due to its lower NaOH requirements. While PCG demand remains relatively constant at lower gas flow rates (Figure S5C), increasing the incoming sulfur content results in a decrease in the fraction of sulfur recovered (Figure S5D); on the other hand, increasing the gas flow rate causes PCG consumption to increase proportionately. BS-PCG reaches the most efficient NaOH consumption between 1,000 and 3,000 ppm, depicting a plateau (Figure S5B). The interplay between these operative parameters suggests that 3,000 ppm is the ideal set point, as indicated by the inflection seen in the respective toxicity-impact categories. Under 10,000 m^3^ h^–1^, the balance between PCG and electric consumption for downstream operations allows a lower GWP for BS-PCG.

Figure 4 shows how the fraction of water purged from the scrubber and the distance from the sea jointly affect both environmental and economic indicators in the CS and BS-PCG scenarios. Similarly, Figure S6 illustrates the trends in resource consumption and byproduct generation under the same analyzed conditions. At higher purge fractions, a larger amount of liquid is removed from the system, which lowers the level of accumulation of acid compounds but increases overall WC and wastewater generation. Figure 4A shows that higher purge ratios tend to slightly favor the CS scenario, even though its WC is also increasing. This apparent advantage arises because in the BS-PCG scenario, WC is positively impacted by glycerol purification processes. Consequently, as larger purge fractions reduce glycerol demand, the beneficial effect associated with glycerol purification diminishes, leading to a more balanced outcome between the two scenarios.

Figure 4B illustrates the implications for operational costs when varying effluent transport to the sea is considered. The analysis reveals that the transport distance significantly influences the comparative operational costs. Specifically, shorter transport distances notably favor the CS scenario due to reduced logistical costs, while long distances reduce this cost advantage. This indicates that the distance is the primary determinant of operational cost differences between scenarios. Interestingly, in the BS-PCG scenario, the increase in water purging, although potentially reducing the accumulation of valuable byproducts such as biogas and elemental sulfur (as shown in Figure S6), appears to be offset by decreased chemical consumption, mitigating cost variations associated with resource demand fluctuations. Therefore, the distance to the sea can be a crucial parameter in determining the best configuration.

Figure 4 further illustrates that toxicity indicators such as HCT (C) and ecotoxicity (D,E,G) can slightly improve for CS when purge rates linked to dilution increase due to the consequential increase in wastewater transport and freshwater usage; nonetheless, BS-PCG still has a better performance across these category impacts. The results in Figure S6 clarify that although BS-PCG recovers less sulfur for higher purging, this limitation is counterbalanced by its lower overall resource consumption, especially in terms of reduced chemical dosing. In Figure 4H, CS consistently outperforms BS-PCG regarding GWP. This is primarily due to the carbon source footprint during crude glycerol purification, particularly the energy required for heat production by natural gas. Even in scenarios where BS-PCG achieves reductions in certain toxicity impacts, the absence of crude glycerol use in CS ensures that its overall carbon footprint remains lower.

Together, Figures 3, 4, and S5 and S6 demonstrate the importance of accounting for the interactions among purge fraction, distance, sulfur load, and reagent consumption. Although increasing the water purge rate can mitigate some resource-related impacts, higher purge rates also increase water requirements and effluent transport demands. In addition, Table S3 shows that the dominant CAPEX items for BS-PCG (bioreactors) scale linearly with the reactor dimensions, which are strongly related with SO_2_ concentration and gas flow rate, implying that the absolute investment required would be markedly lower at reduced plant sizes. Consequently, while CS retains advantages in costs and moderately in GWP, BS-PCG may exhibit improved performance at medium-to-small plant sizes and stable impacts under the analyzed purging rate range, even though its investment costs remain higher than those of CS, albeit reduced at lower capacities due to the approximately linear scaling of reactor-related CAPEX.

Since modifying the plant’s operational conditions did not seem to quite enhance BS-PCG’s economic performance, a sensitivity analysis of resource and byproduct prices was conducted for the BS-PCG scenario under the baseline comparison conditions. Figure 5 presents the NPV trends (€) in BS-PCG as a function of the prices of natural gas—a byproduct of methane generation—and crude glycerol, the primary resource used in the process. The blue dashed line indicates the price conditions at which CS and BS-PCG achieve equal economic performance in terms of NPV; it serves as a boundary between the green and red areas, representing favorable scenarios for BS-PCG and CS, respectively.

These results indicate that lowering the purchasing costs of purified glycerol, by commercial agreement with the producer, or an increase of natural gas prices can effectively improve the profitability of SONOVA. This even considering that purification costs are not determined by natural gas costs, increasing the solidity of what has been discussed. Biogas production therefore allows a greater resilience of BS-PCG to the volatility of natural gas prices, thus reducing the investment risk and thus improving the economic sustainability of the gas treatment process, provided that the consumption of the C source is also reduced.

## 4 Conclusions

The study evaluated the environmental and economic performance of three off-gas treatment methods: a conventional chemical scrubber (CS) and two bioscrubbers, one utilizing fossil-derived glycerol (BS-PG) and the other purified crude glycerol (BS-PCG). Process simulation, LCA, and LCC methodologies provided comprehensive insights into the sustainability and economic feasibility of each configuration.

The bioscrubber configurations (BS-PG and BS-PCG) promoted circularity by enabling recovery of valuable byproducts, such as biogas and elemental sulfur. Despite these environmental advantages, their economic viability remains limited compared to conventional chemical scrubbing due to higher operational complexity, investment costs, and operational expenditures.

From an environmental perspective, BS-PG displayed the least favorable performance, primarily attributed to the fossil-derived glycerol used, resulting in substantial impacts across nearly all categories. Conversely, BS-PCG demonstrated a more balanced environmental profile, performing better than CS in several categories, mainly due to renewable glycerol utilization and efficient byproduct recovery.

Economically, CS consistently emerged as the most cost-effective solution, owing to its simplicity, reduced resource demand, and lower capital investment requirements. However, the BS-PCG configuration holds considerable economic promise under specific favorable conditions, such as smaller-scale operations, proximity to waste sources, and availability of waste heat for glycerol purification. Sensitivity analyses identified the transport distance and water purge rates as influential operational parameters, suggesting that their optimization could enhance economic outcomes.

Overall, the BS-PCG bioscrubber emerges as a promising sustainable gas treatment alternative, provided economic constraints—particularly the cost of purified glycerol—are effectively addressed. Future studies should explore cost-effective glycerol sourcing, opportunities for waste heat integration, and a more detailed, site-specific modeling of purged brine discharge and its marine ecotoxicity implications, to further improve the competitiveness and sustainability of bioscrubber technologies.

## Supplementary Material



## Notes

Detailed model equations, calibration and validation results, process inputs, economic parameters, and complete life-cycle impact assessment data for the bioscrubbers and reference chemical scrubber scenarios, presented as tables and figures (PDF)

## ∥

First author present address: alessio.castagnoli@isprambiente.it
