# Supplementary material for: Assessing the Feasibility of Bioscrubbing for Flue Gas Treatment and Sulfur Recovery: A Comparative Study Using Mathematical Modeling, Life Cycle Analysis, and Life Cycle Costing
Source: ACS Environ Au. 2025 Dec 22;6(2):247–60. doi: 10.1021/acsenvironau.5c00216 (PMC13003355; doi:10.1021/acsenvironau.5c00216)
Supplement: Supplementary file 1 [file vg5c00216_si_001.pdf]

Assessing the feasibility of bioscrubbing for flue gas treatment and sulfur recovery: a comparative study using mathematical modelling, Life Cycle Analysis, and Life Cycle Costing

Alessio Castagnoli[a#], Eric Valdés[b], Francesco Pasciucco[a], Isabella Pecorini[a], Daniel González Alé[b], Giulio Munz[c], David Gabriel[b*]

[a] Department of Energy, Systems Territory and Construction Engineering, University of Pisa, Via C.F. Gabba 22, Tuscany, Pisa, 56122, Italy

[b] GENOCOV Research group, Department of Chemical, Biological and Environmental Engineering, Escola d'Enginyeria, Universitat Autònoma de Barcelona, 08193, Bellaterra, Spain

[c] Department of Civil and Environmental Engineering, University of Florence, Via di S. Marta, 3, 50139, Firenze, Italy

* Email: David.Gabriel@uab.cat

[#]First author present address: alessio.castagnoli@isprambiente.it

A.C.: Italian Institute for Environmental Protection and Research, Via del Cedro 38, Livorno, 57122, Italy

Supplementary material

SM1. Mathematical model of the SONOVA bioscrubber

SM1.1. pH modelling

The concentration of protons S_{H+} was calculated according to the algebraic equation proposed by Jeppsson et al. (2006) (see Eq. S1).

$$S_{H+}=-\frac{\theta}{2}+\frac{1}{2}\cdot\sqrt{\theta^2+4\cdot K_W} \qquad \text{Eq. (S1)}$$

Where K_W is the water ionization constant [M], and θ is the charge balance of ionic species in the liquid phase. To calculate the equilibrium of ionic species, process rates need to be considered for every acid-base pair:

$$r_{AB,j}=K_{AB,j}\cdot(S_{j^-}\cdot S_{H^+} - K_{a,j}\cdot S_{Hj}) \qquad \text{Eq. (S2)}$$

Where $K_{AB,j}$ is the acid-base rate constant [M-1 d-1], set to be 1e10, S_{j^-} is the base concentration [M], $K_{a,j}$ is the acid-base ionization constant [M] and S_{Hj} is the acid concentration [M]. These rates are set to be sufficiently rapid to simulate the fast chemical equilibriums in the liquid phase.

Additionally, a pH control sub-routine was added to the model to maintain a specified setpoint at the UASB inlet and within the CSTR. This was achieved by calculating the charge balance at the target pH by means of Eq. (S3):

$$\theta_{sp}=\frac{K_W - S_{H+sp}{}^2}{S_{H+sp}} \qquad \text{Eq. (S3)}$$

Where S_{H+sp} is the proton concentration at the setpoint pH, which can be calculated as: $S_{H+sp} = 10^{-pH_{sp}}$

The required acid or base concentration is then determined as the difference between the setpoint charge balance and the inlet charge balance θ_{in}:

$[Base]_{add}$=max $(0, \theta_{sp} - \theta_{in})$ Eq. (S4)

$[Acid]_{add}$=max $(0, \theta_{in} - \theta_{sp})$ Eq. (S5)

SM1.2. Physical model of the chemical scrubber

To determine the liquid-phase mass transfer coefficient (k_L), the empirical correlation for spray scrubbers proposed by Amokrane et al. (1994) was applied, as shown in Equation (S6):

$$kL_i = w \cdot \sqrt{\frac{D_i \cdot u^*_i}{d}} \quad \text{Eq. (S6)}$$

Where w is an empirical coefficient [-], D_i is the diffusion coefficient for compound *i*, d is the droplet diameter and u^*_i is defined as:

$$u^*_i = U \sqrt{\frac{c_f \cdot \rho_i}{\rho_w}} \quad \text{Eq. (S7)}$$

where U represents the liquid velocity through the scrubber [m h^{-1}], c_f is a drag coefficient [-], ρ_i is the density of the gaseous compound [kg m^{-3}], and ρ_w is the liquid density, assumed to be 998 kg m^{-3}.

Model calibration and validation

To accurately estimate the key uncertain parameters of the model, the experimental dataset from Guimerà et al. (2020) was used. That study investigated SO_2 and NO_2 absorption in a lab-scale spray scrubber with a working volume of 0.82 L. The gas diffuser

generated atomized droplets of 100 μm, making the number of vertical stages (nvs_a) and the dragging coefficient (c_f) the only uncertain model parameters.

Model calibration was performed using the **fminsearch** function, which iterates over a predefined range of parameter values to minimize an objective function. In this case, the objective function was defined as the average relative root mean squared error (RMSE) for pH and SO_2 outlet concentration in the gas phase, taking the values at steady state for each experiment. 31 experiments were used for calibration, in which the changing conditions were: empty bed retention time (n_1 to n_5), inlet SO_2 concentration (n_6 to n_{11}), initial pH (n_{12} to n_{16}), liquid/gas ratio (n_{17} to n_{21}), sulfate concentration (n_{22} to n_{25}) and gas temperature (n_{26} to n_{31}).
Figure S1 presents the calibration results, comparing experimental data (dots) with model predictions (lines) for pH in the outlet liquid stream and SO_2 in the outlet gas stream.

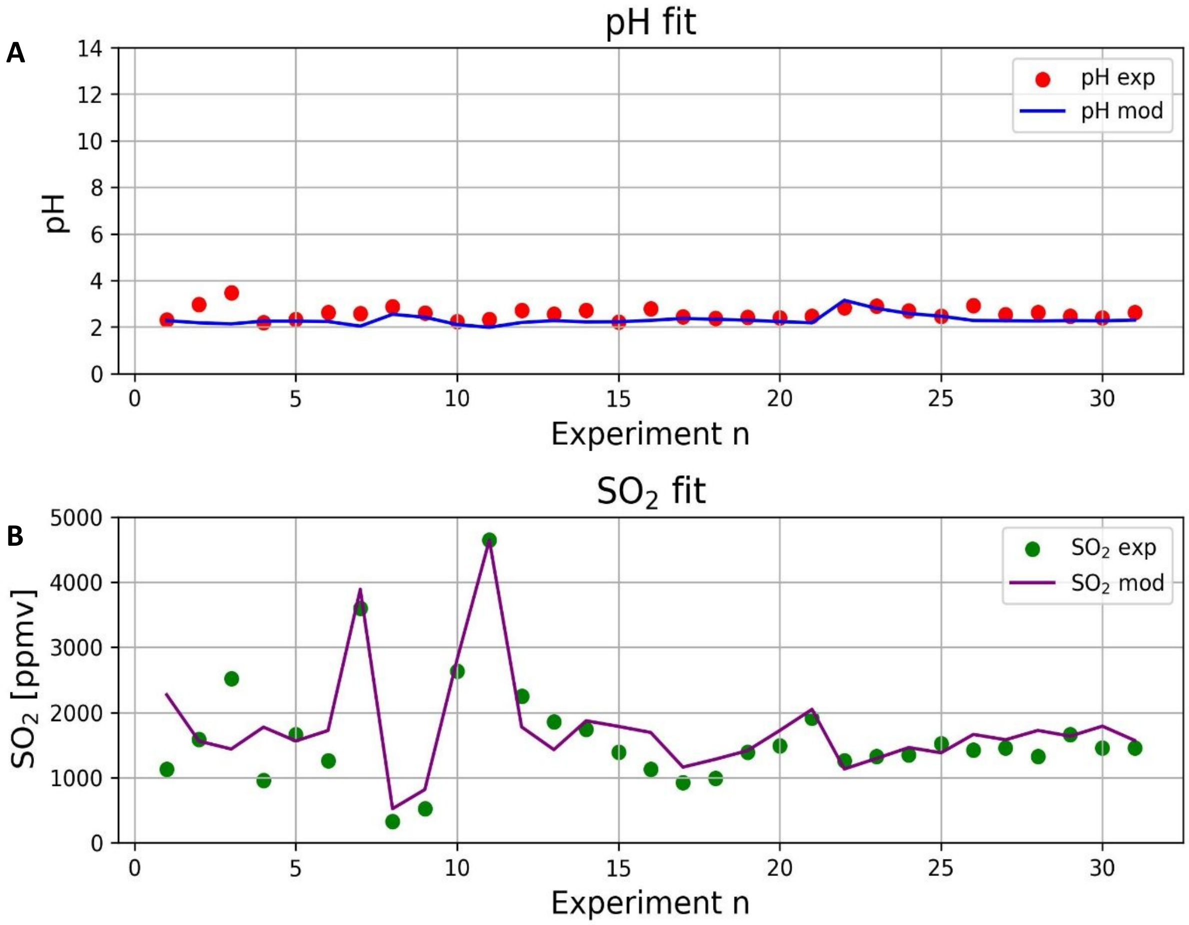

Figure S1. Model calibration results. Dots represent the experimental values of pH (A) and SO_2 concentration in the off gas (B) at the steady state of each experiment, whereas lines represent the model predictions.

During parametric calibration, the objective function was optimized to an average RMSE of $f = 0.11$, and the optimized values were:

- $nvs_a = 2$
- $c_f = 2.47e\text{-}6$

Figure S2 illustrates the strong agreement between experimental and simulated values across the different experimental conditions, confirming the effectiveness of the calibration procedure.

After model calibration, the model was tested against another dataset from the same study where, in addition, CO_2 stripping was also tested by means of dissolving inorganic carbon in the inlet liquid stream. In this case, 10 different experiment datasets with changing inlet of SO_2 concentration in the gas stream were used. Figure S2 shows the experimental values of those experiments along with the estimations of the calibrated model for pH, SO_2 and CO_2 outlet concentrations in the gas phase.

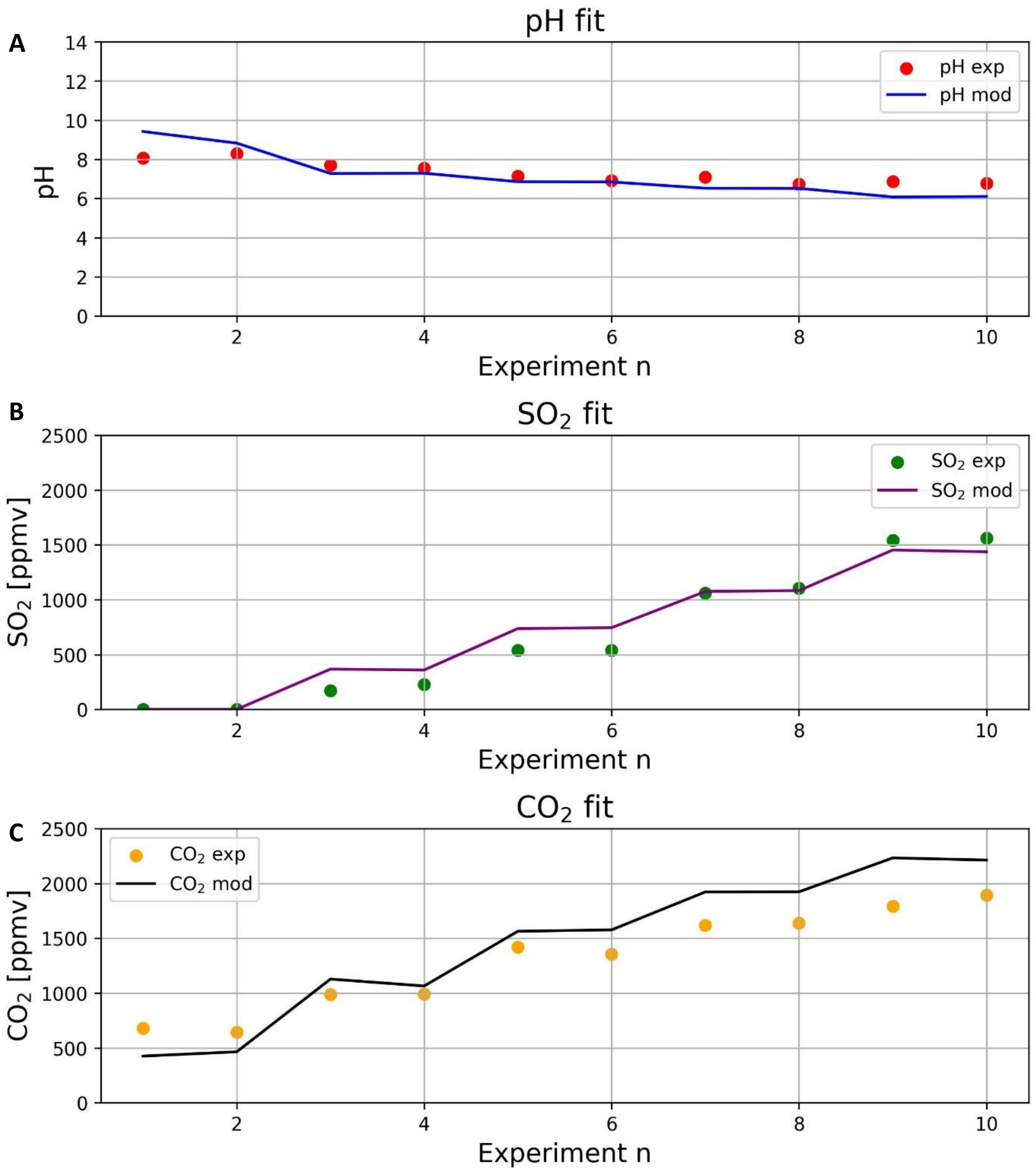

Figure S2. Model validation results. Dots represent the experimental values of pH (A), SO_2 concentration in the off gas (B) and CO_2 concentration in the off gas (C) at the steady state of each experiment, whereas lines represent the model predictions.

In this case, the average relative RMSE was even lower than that of the calibration phase, with a value of f = 0.04. The simulated values show a slight overestimation of CO_2 stripping in the column. However, the primary focus of the study—sulfur absorption—was accurately captured by the model. It is important to note that certain key parameters

affecting mass transfer, such as gas temperature, were not properly monitored during these experiments.

SM1.3. Mathematical model of a mixed tank bioreactor for sulfide oxidation

Biological model

The biological model of the CSTR for sulfide oxidation was formulated according to two already-published models: Mora et al. (2016) for the aerobic sulfide oxidation processes and Activated Sludge Model number 2 (ASM2) for the biodegradation of organic matter [5]. The full sulfur oxidation reactions included three electron donors – sulfide, elemental sulfur and thiosulfate –, and their full stoichiometric equations considering biomass growth are shown below (Eq. S8 to S10):

$$0.5H_2S + 0.5HS^- + 0.42O_2 + 0.075CO_2 + 0.005HCO_3^- + 0.016NH_4^+ + 0.489H^+ \xrightarrow{r_{S^{2-}}} 0.016C_5H_7NO_2 + S^0 + 0.973H_2O \quad \text{Eq. (S8)}$$

$$S^0 + 1.22O_2 + 0.267CO_2 + 0.018HCO_3^- + 0.057NH_4^+ + 1.1H_2O \xrightarrow{r_{S^0}} 0.057C_5H_7NO_2 + SO_4^{2-} + 2.04H^+ \quad \text{Eq. (S9)}$$

$$S_2O_3^{2-} + 1.65O_2 + 0.333CO_2 + 0.022HCO_3^- + 0.071NH_4^+ + 1.12H_2O \xrightarrow{r_{S_2O_3^{2-}}} 0.071C_5H_7NO_2 + SO_4^{2-} + 2.05H^+ \quad \text{Eq. (S10)}$$

Analogously, the modelled pathways for organic matter biodegradation accounted for three forms of COD available for heterotrophic uptake: fermentation products (S_A), fermentable substrates (S_F), and particulate slowly biodegradable substrates (X_S).

These fractions determine the growth and decay processes of heterotrophic biomass, as illustrated in the figure below.

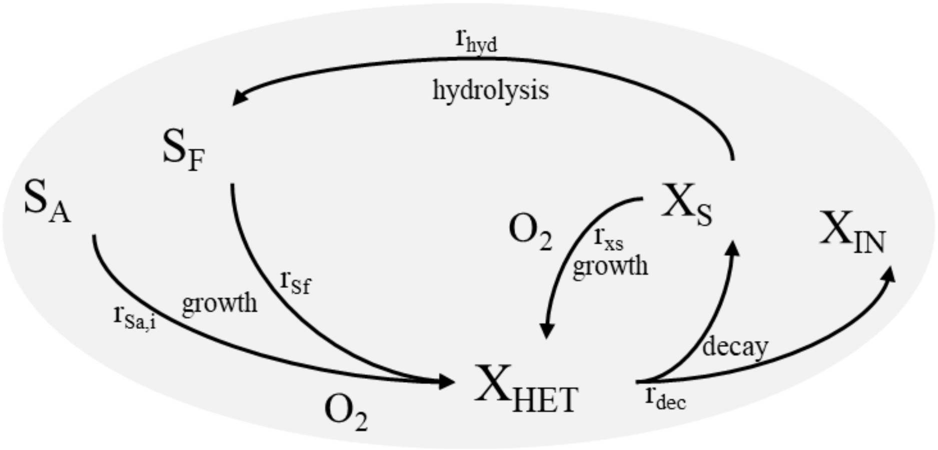

Figure S3. Schematic representation of the COD uptake/production pathways from the activated sludge model number 2 (ADM2), integrated and adapted for this work.

All the biomass decay processes were assumed to generate X_S and particulate inerts (X_{IN}) in proportions of 90% and 10%, respectively.

It is important to note that in the SONOVA bioscrubber model many C compounds are generated from glycerol fermentation processes; all the produced VFAs and alcohols – acetate, propionate, formate, 3-hydroxypropionate, 1,3-propanediol and ethanol – were classified as S_A, while glycerol was considered the only fermentable substrate.

Control of DO

Additionally, DO control was required to calculate the air supply needed in the CSTR, and this was achieved by means of a proportional integral (PI) control. This control method adjusts the controller output based on the error and the integral of the error by means of Eq. (S11):

$$c(t) = K_c \cdot \left(e(t) + \frac{1}{Ti} \cdot \int e(t)dt \right) + C \qquad \text{Eq. (S11)}$$

Where $c(t)$ is the controller output – in this case the air flowrate supplied [$m^3\ h^{-1}$] –, K_c is the controller gain, Ti is the time integral, $e(t)$ is the error – in this case denoted as $DO - DO_{sp}$ – and C is the initial value of the controller.

The air flowrate and the volumetric mass transfer coefficient of oxygen ($k_L a$) in the CSTR were linked with a correlation for activated sludge in cylindrical aerated mixed tanks proposed by Pittoors et al. (2014):

$$k_L a = \left(\frac{0.06 \cdot D}{{D_t}^2}\right) \cdot Re^{1.906} \cdot Fr^{-0.631} \cdot \left(\frac{d_b}{h_d}\right)^{-0.23} \cdot \left(\frac{H_t}{D_t}\right)^{-0.12} \quad \text{Eq. (S12)}$$

$$\cdot \left(\frac{A_d}{A_t}\right)^{0.326} \cdot \left(\frac{D_t}{h_d}\right)^{0.164} \cdot \left(\frac{H_t}{h_d}\right)^{0.173} \cdot \left(\frac{V_t}{{A_d}^{1.5}}\right)^{-0.01}$$

Where the Reynolds number is calculated as $\left(Re = \frac{Qa \cdot \rho}{D\eta}\right)$, the Froude number is calculated as $\left(Fr = \frac{Qa}{\sqrt{{D_t}^5 \cdot g}}\right)$, D is the diffusion coefficient of oxygen [m^2 s^{-1}], D_t is the tank diameter [m], d_b is the bubble diameter [m], h_d is the diffuser submergence [m], H_t is the height of the tank [m], A_d is the total coverage area of the diffusers [m^2], A_t is the total area of the tank [m^2] and V_t is the volume of the tank [m^3].

SM1.4 . Supplementary equations for energy consumption

The % of utilized energy is a function of the inlet conditions of the process as well as the biosulfur recovery capacity of the biological stages, and can be calculated by means of Eq. (S13):

$$\%E_{ut} = 100 \cdot \left(1 - \frac{Q_{gas} - Q_{dry} - Q_{bio}}{Q_{gas}}\right) \quad \text{Eq. (S13)}$$

Where Q_{gas} is the available energy from the combustion gases, Q_{dry} is the required energy for sludge drying and Q_{bio} is the energy needed for temperature control in the biological units [kJ h^{-1}].

The pumps are assumed to operate under inverter control, meaning their energy consumption is proportional to the actual flow rate relative to the nominal flow rate

provided by the pump's datasheet. Thus, the energy consumption of the pump (E_{pump}, expressed in kWh) is calculated using the following equation:

$$E_{pump} = P_{nom} * \frac{Q_{nom}}{Q_{mod}} * t \qquad \text{Eq. (S14)}$$

Where P_{nom} [kW] and Q_{nom} [m^3 h^{-1}] are the nominal power and flowrate of the pump (obtained from data sheet), Q_{mod} [m^3 h^{-1}] is the model flowrate and t is the operational time considered [h].

The energy consumption of centrifuges (E_{centr}, expressed in kWh h^{-1}) used for sludge dewatering is estimated based on the specific energy required to remove volatile suspended solids (VSS):

$$E_{centr} = E_{spec} * VSS_{in} * \eta * t \qquad \text{Eq. (S15)}$$

Where VSS_{in} [kg h^{-1}] is the Volatile Suspended Solids (VSS) mass flow, η is the operational efficiency (yield, assumed to be 0.8), E_{spec} [kWh kgVSS^{-1}] is the specific energy needed for centrifuge 1 kg of VSS assumed as 0.5.

SM2. Figures and tables

Table S1. List of inputs for the chemical scrubber (CS) – SONOVA bioscrubber (BS-PG and BS-PCG) mathematical models.

	Parameter	Units	CS	BS-PG	BS-PCG
**Flowrates**	Gas	$m^3 h^{-1}$	32000	32000	32000
	Gas/liquid ratio	$m^3 h^{-1}$	145,45	145,45	145,45
	Liquid purge	%	1	1	1
	Water temperature	ºC	25	25	25
	River water pH	-	8,1	8,1	8,1
	Air temperature	ºC	20	20	20
**Chemical absorber**	HRT	s	0,6	0,6	0,6
	P inlet	atm	0,9	0,9	0,9
	P outlet	atm	1	1	1
	T inlet	ºC	80	80	80
	T outlet	ºC	34	34	34
	SO_2 concentration	ppmv	3120	3120	3120
	CO_2 concentration	ppmv	3200	3200	3200
	Inlet pH	--	6	6	6
	Spray tower (1=yes, 0=no)	-	1	1	1
	Packed tower (1=yes, 0=no)	-	0	0	0
	Type of packing ("num code")	-	0	0	0
	Droplet diameter	mm	0,18	0,18	0,18
	Number of sections		2	2	2
	Height/diameter ratio		1	1	1
	Holdup (1% for ST; 10% for PT)	%	1	1	1
**UASB bioreactor**	HRT	h	4,4	4,4	4,4
	C/S ratio	mg C mg S^{-1}	1,6	1,6	1,6
	Glycerol purity	%	0	100	95

**for sulfate reduction (UASB SRB)**	Impurities concentration	%	0	0	0
	Inlet pH	--	8	8	8
	Number of sections		3	3	3
	Height/diameter ratio	-	2	2	2
	Headspace % top section	%	10	10	10
	Liquid temperature	ºC	35	35	35
**Aerated mixed tank bioreactor for sulfide oxidation (pSOB)**	HRT	h	9	9	9
	DO setpoint	mg O_2 L^{-1}	0,1	0,1	0,1
	pH	--	6	6	6
	Height/diameter ratio	-	1	1	1
	Headspace %		15	15	15
	Liquid temperature	ºC	35	35	35

Table S2. List of Ecoinvent processes used for LCIA modeling

**Flow**	**Ecoinvent process**
Freshwater	Tap water {RER}| market group for tap water | APOS, U
NaOH	Sodium hydroxide, without water, in 50% solution state {RER}| market for sodium hydroxide, without water, in 50% solution state | APOS, U
Glycerine	Glycerine {RER}| market for glycerine | APOS, U
Energy	Electricity, medium voltage {RER}| market group for electricity, medium voltage | APOS, U
Voltage transformation	Electricity, low voltage {ES}| electricity voltage transformation from medium to low voltage | APOS, U
Transports	Transport, freight, lorry >32 metric ton, EURO6 {RER}| market for transport, freight, lorry >32 metric ton, EURO6 | APOS, U
CO_2 emission (biogenic)	Carbon dioxide, biogenic
CO_2 emission (fossil)	Carbon dioxide, fossil
SO_2 emissions	Sulfur dioxide, ES

Biogas production (system expansion)	Natural gas, high pressure {RoW}| market for natural gas, high pressure | APOS, U
Sulfur production (system expansion)	Sulfur {GLO}| market for sulfur | APOS, U
Purified glycerol process	
Hydrochloric Acid	Hydrochloric acid, without water, in 30% solution state {RER}| market for hydrochloric acid, without water, in 30% solution state | APOS, U
High voltage electricity	Electricity, high voltage {NL}| market for electricity, high voltage | APOS, U
Steam	Heat, district or industrial, natural gas {Europe without Switzerland}| heat production, natural gas, at boiler modulating >100kW | APOS, U
Market transports	Transport, freight train {RER}| market group for transport, freight train | APOS, U
Cooling sludge disposed	Sewage sludge, 97% water, WWT-SLF, residue from cooling tower {CH}| market for sewage sludge, 97% water, WWT-SLF, residue from cooling tower | APOS, U
Salt disposed by landfill	Salt tailing from potash mine {RER}| treatment of salt tailing from potash mine, residual material landfill | APOS, U
Solvent mixture incinerated	Spent solvent mixture {Europe without Switzerland}| treatment of spent solvent mixture, hazardous waste incineration, with energy recovery | APOS, U
Wastewater treated	Wastewater, average {Europe without Switzerland}| market for wastewater, average | APOS, U

.

Table S3. Relative CAPEX costs of the all components included in system boundaries

Component	Unit	CS	BS-PG	BS-PCG	Year
UASB bioreactor	€ m^{-3}	0	650	650	2024
pSOB bioreactor	€ m^{-3}	0	600	600	2024
Absorption tower	€ m^{-3} h^{-1}	0.2	0.2	0.2	2024
Absorption tower - fixed costs	€	3000	3000	3000	2024
Gasometer	€ m^{-3} h^{-1}	0	18.5	18.5	2024
Instrumentation for biogas analysis	€	0	56	56	2024
Centrifuge	€ m^{-3} h^{-1}	0	490	490	2024
S dryer	€ m^{-3} h^{-1}	0	2000	2000	2024
Piping and installation	% of reactor cost	30%	30%	30%	2024
Instrumentation	€	12000	35000	35000	2024
Recirc pump Absorber	€ m^{-3} h^{-1}	93	93	93	2024
pH and redox control (with dosing included)	€	7000	7000	7000	2024
Pressure drop measuring system	€	200	200	200	2024
Demister	€	1000	64	64	2024
Discharge pump absorber	€ m^{-3} h^{-1}	93	93	93	2024
Fresh water supply pump	€ m^{-3} h^{-1}	150	93	93	2024
Polyelectrolyte pump	€ m^{-3} h^{-1}	0	50	50	2024
Glycerol/Glycerine feeding pump	€ m^{-3} h^{-1}	0	93	93	2024
Construction of remote control building and changing room	€	0	25000	25000	2024
Electrical substation construction	€	0	25000	25000	2024
Arrangement and implementation of PLC and electrical system	€	6000	15000	15000	2024
Carpentry works	€	5000	15000	15000	2024
Design, construction management, safety during the design and construction phase	€	5000	10000	10000	2024
Costs for authorization	€	2000	5000	5000	2024
Laboratory equipment and tools	€	500	3000	3000	2024
Centrigue feeding pump	€ m^{-3} h^{-1}	0	93	93	2024
External SONOVA Recirculation pump	€ m^{-3} h^{-1}	0	93	93	2024

Dryer feeding pump	€ m^{-3} h^{-1}	0	93	93	2024
Dryer blower - fixed costs	€	0	1047	1047	2024
Dryer blower	€ m^{-3} h^{-1}	0	7.1	7.1	2024
Aeration blower CSTR - fixed costs	€	0	1047	1047	2024
Aeration blower CSTR	€ m^{-3} h^{-1}	0	7.1	7.1	2024
Water heater/Heat exchanger - fixed costs	€	0	32200	32200	2024
Water heater/Heat exchanger	€ W^{-1}	0	32.4	32.4	2024
Reactors heating system pump	€ W^{-1}	0	0.035	0.035	2024

Table S4. Relative OPEX related to the system (labour excluded)

Parameters	Unit	Average	Glycerine	Glycerol	Year
NaOH 50%	€ tn^{-1}	527	527	527	2021
Glicerine	€ tn^{-1}	0	745	NN	2021
Purified glycerol	€ tn^{-1}	0	NN	520	2020
Sulfur	€ tn^{-1}	150	150	150	2021
Electricity	€ kWh^{-1}	0.088	0.088	0.088	2021 (Eurostat)
Fresh water	€ m^{-3}	0.06	0.06	0.06	2021 (industry cost)
Natural gas	€ m^{-3}	0.354	0.354	0.354	2021 (Eurostat)
Water disposal transportation	€ m^{-3}	7.23	7.23	7.23	2021 (industry cost)
UASB SRB bioreactor - maintainance	% capex costs y^{-1}	0	5%	5%	
pSOB bioreactor - maintainance	% capex costs y^{-1}	0	5%	5%	
Absorption tower and piping - maintainance	% capex costs y^{-1}	2%	2%	2%	
Gasometer - maintainance	% capex costs y^{-1}	0	5%	5%	
Centrifuge - maintainance	% capex costs y^{-1}	0	5%	5%	
Recirc pump Absorber - maintainance	% capex costs y^{-1}	10%	5%	5%	
pH and redox control - maintainance	% capex costs y^{-1}	10%	10%	10%	
Discharge pump absorber - maintainance	% capex costs y^{-1}	10%	10%	10%	
Fresh water supply pump maintainance	% capex costs y^{-1}	10%	10%	10%	

Polyelectrolyte pump - maintainance	% capex costs y^{-1}	0	10%	10%	
Glycerol/Glycerine feeding pump - maintainance	% capex costs y^{-1}	0	0.1	10%	
Centrigue feeding pump - maintainance	% capex costs y^{-1}	0	0.1	10%	
External SONOVA Recirculation pump - maintainance	% capex costs y^{-1}	0	0.1	10%	
Dryer feeding pump - maintainance	% capex costs y^{-1}	0	0.1	10%	
Dryer blower - maintainance	% capex costs y^{-1}	0	0.1	10%	
Aeration blower CSTR - maintainance	% capex costs y^{-1}	0	0.1	10%	
Heat exchanger - maintainance	% capex costs y^{-1}	0	0.1	10%	
Reactors heating system pump - maintainance	% capex costs y^{-1}	0	0.1	10%	

Table S5. Life Cycle Impact Assessment results for CS, BS-PG and BS-PCG scenarios

Impact category	CS	BS-PG	BS-PCG
Global warming (kgCO_{2eq})	1,378,946.35	7,323,335.63	1,644,857.49
Stratospheric ozone depletion (kg $CFC11_{eq}$)	0.72	39.70	0.75
Ionizing radiation (kBq Co-60_{eq})	370,209.92	696,858.51	394,262.47
Ozone formation, Human health (kg NOx_{eq})	2748.97	16,887.61	2115.13
Fine particulate matter formation (kg $PM2.5_{eq}$)	1721.72	10,190.31	-855.63
Ozone formation, Terrestrial ecosystems (kg NOx_{eq})	2871.40	17,617.19	2153.07
Terrestrial acidification (kg SO_{2eq})	4124.24	44,420.47	-4214.68
Freshwater eutrophication (kg P_{eq})	1056.73	3168.74	900.94
Marine eutrophication (kg N_{eq})	147.98	15,089.73	261.76
Terrestrial ecotoxicity (kg 1,4-DCB)	18,903,701.29	65,305,633.75	10,888,868.46
Freshwater ecotoxicity (kg 1,4-DCB)	120,618.56	375,341.97	97,568.01
Marine ecotoxicity (kg 1,4-DCB)	174,882.17	544,760.63	139,492.30
Human carcinogenic toxicity (kg 1,4-DCB)	384,168.46	1,308,197.03	298,268.28
Human non-carcinogenic toxicity (kg 1,4-DCB)	2,528,013.03	15,480,322.16	2,471,435.58
Land use (m^2a $crop_{eq}$)	76,207.95	15,953,332.99	63,443.81
Mineral resource scarcity (kg CU_{eq})	7460.58	27,542.89	5960.66
Fossil resource scarcity (kg oil_{eq})	376,962.60	1,633,997.61	192,451.96
Water consumption (m^3)	47,431.03	172,131.33	20,255.39

Table S6. Life Cycle Impact Assessment Normalized Results for CS, BS-PG and BS-PCG scenarios

**Category of Impact**	**U.M**	**CS**	**BS-PG**	**BS-PCG**
Global warming	PE	158.98	1758.79	348.97
Stratospheric ozone depletion	PE	11.06	1236.52	20.48
Ionizing radiation	PE	713.97	2265.65	1088.98
Ozone formation, Human health	PE	123.20	1507.84	165.66
Fine particulate matter formation	PE	61.94	828.84	21.42
Ozone formation, Terrestrial ecosystems	PE	149.17	1822.06	194.43
Terrestrial acidification	PE	92.53	2213.77	-4.73
Freshwater eutrophication	PE	1490.13	8797.63	2268.64
Marine eutrophication	PE	29.37	6116.33	100.98
Terrestrial ecotoxicity	PE	1157.12	8004.03	1310.14
Freshwater ecotoxicity	PE	4384.75	27010.72	6394.80
Marine ecotoxicity	PE	3688.98	22708.90	5283.17
Human carcinogenic toxicity	PE	34,408.15	229,744.79	46,416.12
Human non-carcinogenic toxicity	PE	74.21	909.20	130.97
Land use	PE	11.41	4828.15	15.81
Mineral resource scarcity	PE	0.06	0.42	0.08
Fossil resource scarcity	PE	355.32	3045.11	296.27
Water consumption	PE	170.53	1103.94	39.21

Table S7. Investment costs for the equipment

	Unit of measure	CS	BS-PG	BS-PCG
UASB SRB bioreactor	€	0.00	-1,476,605.52	-1,476,605.52
pSOB bioreactor	€	0.00	-598,763.26	-598,763.26
Absorption tower	€	-0.73	-0.73	-0.73
Absorption tower - fixed costs	€	-3000.00	-3000.00	-3000.00
Gasometer	€	0.00	-813.42	-813.42
Instrumentation for biogas analysis	€	0.00	-56.00	-56.00
Centrifuge	€	0.00	-147,000.00	-147,000.00
S dryer	€	0.00	-152,983.88	-152,983.88
Piping and installation	€	-900.22	-623,510.85	-623,510.85
Recirc pump Absorber	€	-20,460.00	-20,460.00	-20,460.00
pH and redox control (with dosing included)	€	-7000.00	-7000.00	-7000.00
Pressure drop measuring system	€	-200.00	-200.00	-200.00
Demister	€	-1000.00	-64.00	-64.00
Discharge pump absorber	€	-204.60	-204.60	-204.60
Fresh water supply pump	€	-406.52	-261.95	-261.95
Polyelectrolyte pump	€	0.00	0.00	0.00
Glycerol/Glycerine feeding pump	€	0.00	-18.43	-19.40
Construction of remote control building and changing room	€	0.00	-25,000.00	-25,000.00
Electrical substation construction	€	0.00	-25,000.00	-25,000.00
Arrangement and implementation of PLC and electrical system	€	-6000.00	-15,000.00	-15,000.00
Carpentry works	€	-5000.00	-15,000.00	-15,000.00
Design, construction management, safety during the design and construction phase	€	-5000.00	-10,000.00	-10,000.00
Costs for authorization	€	-2000.00	-5000.00	-5000.00
Laboratory equipment and tools	€	-500.00	-3000.00	-3000.00
Centrigue feeding pump	€	0.00	-20,412.38	-20,412.38

Dryer feeding pump	€	0.00	-7113.75	-7113.75
Dryer blower	€	0.00	-1590.09	-1590.09
Aeration blower CSTR	€	0.00	-1061.76	-1061.76
Water heater/Heat exchanger	€	0.00	-32,200.00	-32,200.00
Total	€	-51,672.07	-3,044,320.64	-3,044,321.61

Table S8. Annual mantaining costs for the equipment

	Unit of measure	CS	BS-PG	BS-PCG
**UASB SRB bioreactor**	€ y^{-1}	0.00	-74,002.50	-74,002.50
**pSOB bioreactor**	€ y^{-1}	0.00	-30,008.00	-30,008.00
**Absorption tower and piping**	€ y^{-1}	-78.02	-12,559.28	-12,559.28
**Gasometer**	€ y^{-1}	0.00	-73.85	-73.85
**Centrifuge**	€ y^{-1}	0.00	0.00	0.00
**Recirc pump Absorber**	€ y^{-1}	-2,046.00	-1,023.00	-1,023.00
**pH and redox control**	€ y^{-1}	-700.00	-700.00	-700.00
**Discharge pump absorber**	€ y^{-1}	-20.46	-20.46	-20.46
**Fresh water supply pump**	€ y^{-1}	-40.65	-26.16	-26.16
**Polyelectrolyte pump**	€ y^{-1}	0.00	0.00	0.00
**Glycerol/Glycerine feeding pump**	€ y^{-1}	0.00	0.00	0.00
**Centrigue feeding pump**	€ y^{-1}	0.00	-2,046.00	-2,046.00
**External SONOVA Recirculation pump**	€ y^{-1}	0.00	0.00	0.00
**Dryer feeding pump**	€ y^{-1}	0.00	-174.78	-174.78
**Dryer blower**	€ y^{-1}	0.00	-118.04	-118.04
**Aeration blower CSTR**	€ y^{-1}	0.00	-175.70	-175.70
**Heat exchanger**	€ y^{-1}	0.00	-3,220.00	-3,220.00
**Reactors heating system pump**	€ y^{-1}	0.00	0.00	0.00
**Total**	€ y^{-1}	-2,885	-124,148	-124,148

Table S9. Annual labour costs (Eurostat)

		U. M.	Relative weekly presence	Cost per hour (EU)	Cost per person	Annual cost
**CS**	Head of the plant (manager)	€	0.025	28.62	-56,001	-1,400
	Technician or associate professionals	€	0.125	17.76	-34,751	-4,344
	Technician availability outside work schedule	€	0.0125	26.64	-52,126	-652
	**Total**	**€**	**-6,395**			
**BS-PG**	Head of the plant (manager)	€	0.1	28.62	-56,001	-5,600
	Technician or associate professionals	€	0.25	17.76	-34,751	-8,688
	Plant and machine operator and assemblers	€	0.5	11.45	-22,404	-11,202
	Technician availability outside work schedule	€	0.025	26.64	-52,126	-1,303
	Operator availability outside work schedule	€	0.05	17.175	-33,606	-1,680
	**Total**	**€**	**-28,473**			
**BS-PCG**	Head of the plant (manager)	€	0.1	28.62	-56,001	-5,600
	Technician or associate professionals	€	0.25	17.76	-34,751	-8,688
	Plant and machine operator and assemblers	€	0.5	11.45	-22,404	-11,202
	Technician availability outside work schedule	€	0.025	26.64	-52,126	-1,303
	Operator availability outside work schedule	€	0.05	17.175	-33,606	-1,680
	**Total**	**€**	**-28,473**			

Table S10. Annual operative costs

	Unit of measure	CS	BS-PG	BS-PCG
Electric energy	€ y^{-1}	-14,047	-29,479	-29,479
Freshwater	€ y^{-1}	-1,425	-1,480	-1,480
NaOH	€ y^{-1}	-611,167	-648,581	-648,581
C source	€ y^{-1}	0	-3,050,461	-2,241,243
Wastewater transport	€ y^{-1}	-139,432	-139,337	-139,337
Biogas	€ y^{-1}	0	480,764	480,764
Sulfur	€ y^{-1}	0	53,538	53,538
Total	€ y^{-1}	**-766,071**	**-3,335,034**	**-2,525,816**

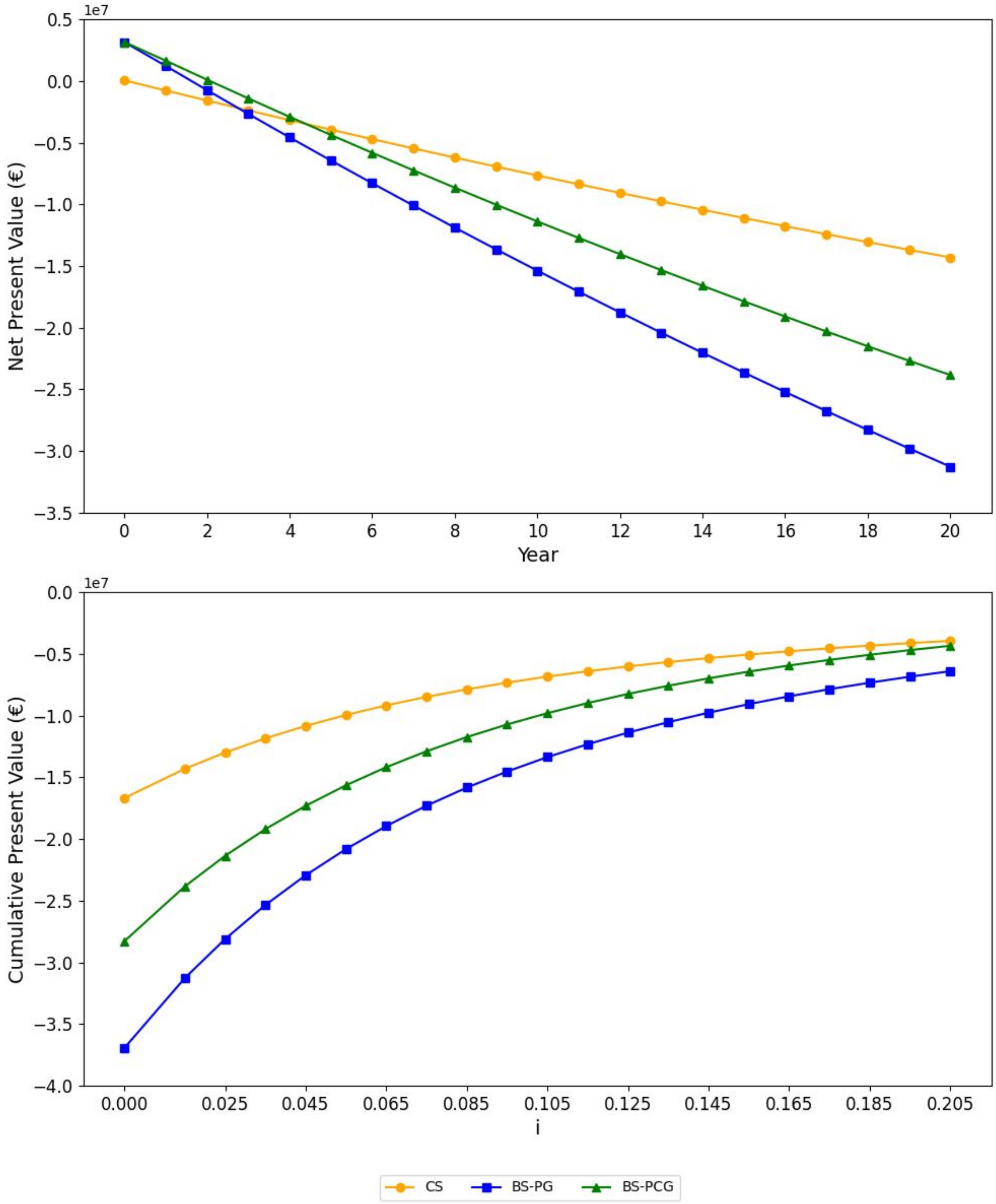

Figure S4. Net Present Value (NPV) trend over the life cycle time and Cumulative Present Value sensitivity analysis on the interest rate (i).

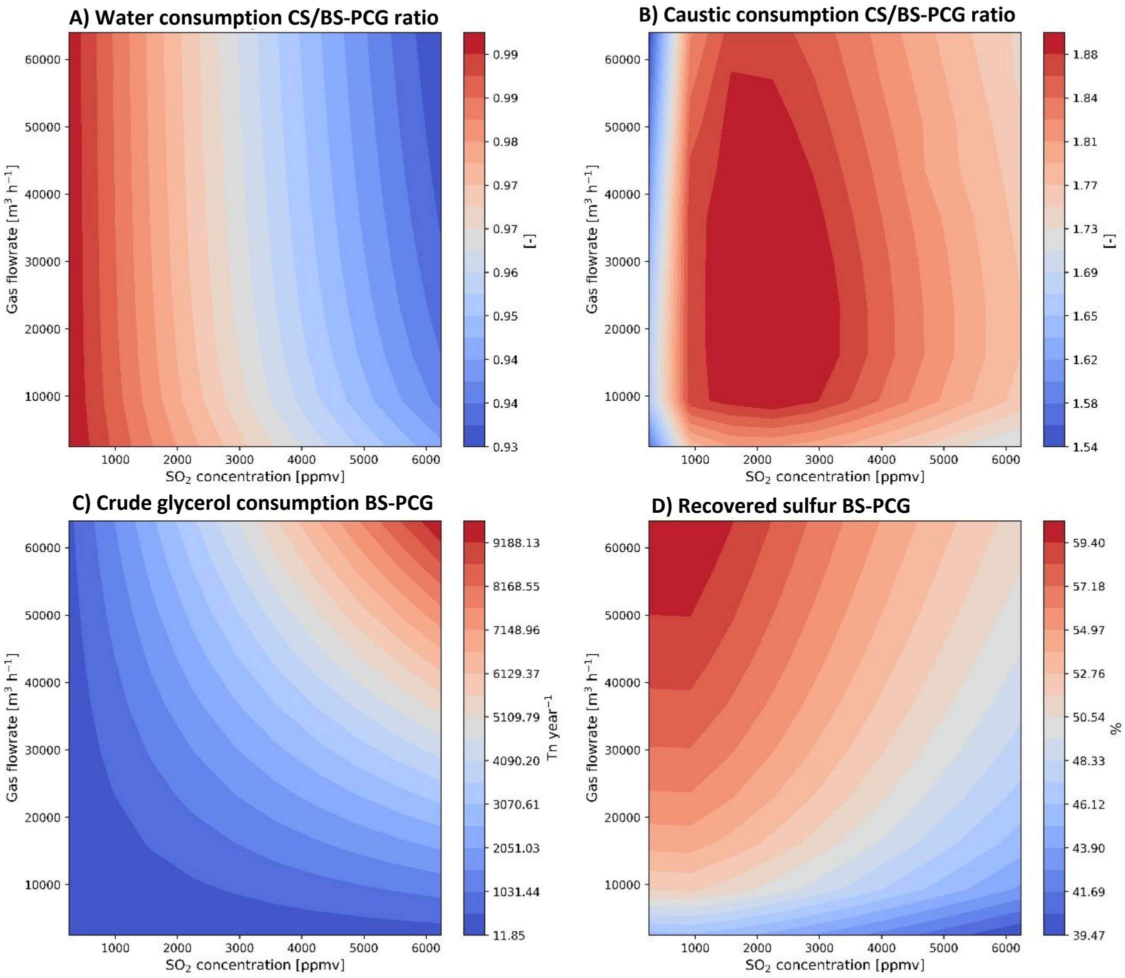

Figure S5. Resources consumption and recovery of byproducts in the analyzed scenarios of section 3.4. of the study, under the effect of inlet gas flowrate and SO_2 concentration. A) Water consumption ratio between CS and BS-PCG (CS/BS-PCG), B) Water consumption ratio between CS and BS-PCG (CS/BS-PCG), C) crude glycerol consumption in BS-PCG [Tn gly y^{-1}] and D) recovered sulfur in BS-PCG expressed as the % of total treated sulfur contained in the emissions are showed.

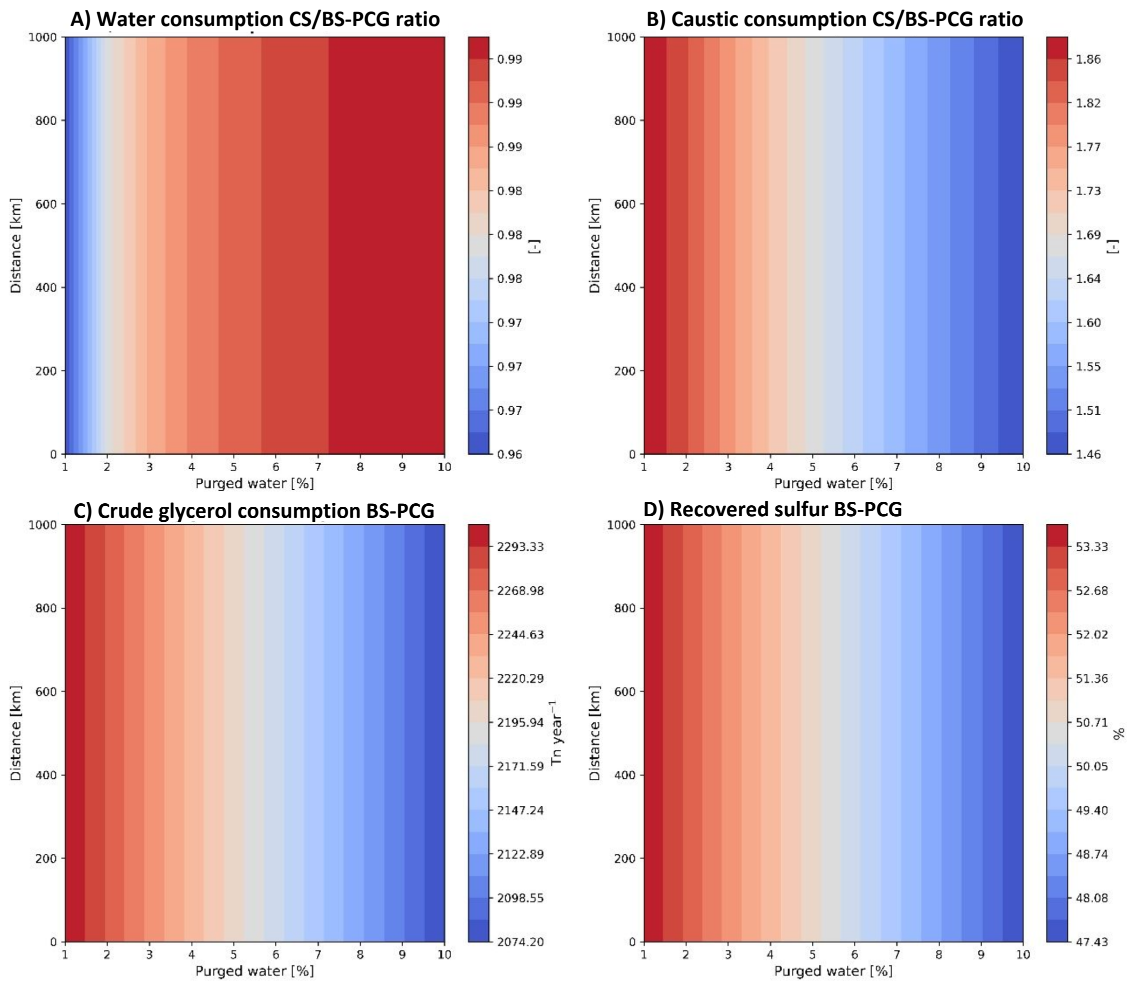

Figure S6. Resources consumption and recovery of byproducts in the analyzed scenarios of section 3.4. of the study, under the effect of purged water and distance of plant to sea. A) Water consumption ratio between CS and BS-PCG (CS/BS-PCG), B) Water consumption ratio between CS and BS-PCG (CS/BS-PCG), C) crude glycerol consumption in BS-PCG [Tn gly y^{-1}] and D) recovered sulfur in BS-PCG expressed as the % of total treated sulfur contained in the emissions are showed.

References

[1] U. Jeppsson, C. Rosen, J. Alex, J. Copp, K.V. Gernaey, M.-N. Pons, P.A. Vanrolleghem, Towards a benchmark simulation model for plant-wide control strategy performance evaluation of WWTPs, Water Sci. Technol. 53 (2006) 287–295. https://doi.org/10.2166/wst.2006.031.

[2] H. Amokrane, A. Saboni, B. Caussade, Experimental study and parameterization of gas absorption by water drops, AIChE J. 40 (1994) 1950–1960. https://doi.org/10.1002/aic.690401204.

[3] X. Guimerà, M. Mora, L.R. López, G. Gabriel, A.D. Dorado, J. Lafuente, X. Gamisans, D. Gabriel, Coupling dissolved oxygen microsensors measurements and heterogeneous respirometry for monitoring and modeling microbial activity within sulfide-oxidizing biofilms, Chem. Eng. J. 400 (2020) 125846. https://doi.org/10.1016/j.cej.2020.125846.

[4] M. Mora, L.R. López, J. Lafuente, J. Pérez, R. Kleerebezem, M.C.M. Van Loosdrecht, X. Gamisans, D. Gabriel, Respirometric characterization of aerobic sulfide, thiosulfate and elemental sulfur oxidation by S-oxidizing biomass, Water Res. 89 (2016) 282–292. https://doi.org/10.1016/j.watres.2015.11.061.

[5] M. Henze, W. Gujer, M. Takahashi, M. Tomonori, M. Wentzel, G. Marais, M. Van Loosdrecht, Activated Sludge Model No.2d, ASM2d, Water Sci. Technol. 39 (1999). https://doi.org/10.1016/S0273-1223(98)00829-4.

[6] E. Pittoors, Y. Guo, S. W. H. Van Hulle, Modeling dissolved oxygen concentration for optimizing aeration systems and reducing oxygen consumption in activated sludge processes: a review, Chem. Eng. Commun. 201 (2014) 983–1002. https://doi.org/10.1080/00986445.2014.883974.

[7] T. Attarbachi, M.D. Kingsley, V. Spallina, New trends on crude glycerol purification: A review, Fuel 340 (2023) 127485. https://doi.org/10.1016/j.fuel.2023.127485.
